# Itaconate facilitates viral infection via alkylating GDI2 and retaining Rab GTPase on the membrane

**DOI:** 10.1038/s41392-024-02077-8

**Published:** 2024-12-27

**Authors:** Shulei Yin, Yijie Tao, Tianliang Li, Chunzhen Li, Yani Cui, Yunyan Zhang, Shenhui Yin, Liyuan Zhao, Panpan Hu, Likun Cui, Yunyang Wu, Yixian He, Shu Yu, Jie Chen, Shaoteng Lu, Guifang Qiu, Mengqi Song, Qianshan Hou, Cheng Qian, Zui Zou, Sheng Xu, Yizhi Yu

**Affiliations:** 1 National Key Laboratory of Immunity and Inflammation, Naval Medical University, Shanghai, 200433 China; 2 School of Anesthesiology, Naval Medical University, Shanghai, 200433 China; 3 Department of Respiratory and Critical Care Medicine, Changzheng Hospital, Naval Medical University, Shanghai, 200433 China; 4 Department of Traditional Chinese Medicine, Naval Medical University, Shanghai, 200433 China; 5 Faculty of Anesthesiology, Changhai Hospital, Naval Medical University, Shanghai, 200433 China

**Keywords:** Infection, Innate immunity

## Abstract

Metabolic reprogramming of host cells plays critical roles during viral infection. Itaconate, a metabolite produced from cis-aconitate in the tricarboxylic acid cycle (TCA) by immune responsive gene 1 (IRG1), is involved in regulating innate immune response and pathogen infection. However, its involvement in viral infection and underlying mechanisms remain incompletely understood. Here, we demonstrate that the IRG1-itaconate axis facilitates the infections of VSV and IAV in macrophages and epithelial cells via Rab GTPases redistribution. Mechanistically, itaconate promotes the retention of Rab GTPases on the membrane via directly alkylating Rab GDP dissociation inhibitor beta (GDI2), the latter of which extracts Rab GTPases from the membrane to the cytoplasm. Multiple alkylated residues by itaconate, including cysteines 203, 335, and 414 on GDI2, were found to be important during viral infection. Additionally, this effect of itaconate needs an adequate distribution of Rab GTPases on the membrane, which relies on Rab geranylgeranyl transferase (GGTase-II)-mediated geranylgeranylation of Rab GTPases. The single-cell RNA sequencing data revealed high expression of IRG1 primarily in neutrophils during viral infection. Co-cultured and in vivo animal experiments demonstrated that itaconate produced by neutrophils plays a dominant role in promoting viral infection. Overall, our study reveals that neutrophils-derived itaconate facilitates viral infection via redistribution of Rab GTPases, suggesting potential targets for antiviral therapy.

## Introduction

Metabolism is the most fundamental mechanism for providing energy and materials for all biological processes, including the life cycle of viruses.^1^ Viral infection triggers drastic metabolic reprogramming, which can substantially impact infectious outcomes.^2^ Viruses have evolved mechanisms to usurp the host’s metabolic resources, funneling them towards the production of virion components, as well as organizing specialized compartments for replication, maturation, and dissemination.^3^ Meanwhile, hosts have developed a variety of metabolic countermeasures to resist viral infection.^4^ However, the complex interplay between viruses and hosts over metabolic control remains incompletely elucidated.

*Irg1* is the encoding gene of mammalian cis-aconitic acid decarboxylase (CAD), also known as *Acod1*. IRG1 converts cis-aconitate, an intermediate of the tricarboxylic acid (TCA) cycle, to itaconate.^5^ Recently, itaconate and its derivatives have attracted extensive attention and research due to their potent immunomodulatory effects targeting various targets, such as Nrf2,^6^ IκBζ^7^ and TET2.^8^ Itaconate and its derivatives containing an electrophilic α, β-unsaturated carboxylic acid can potentially alkylate protein cysteine residues to form a 2,3-dicarboxypropyl adduct, also called “itaconation”.^9^ Itaconation is one of the key mechanisms through which itaconate functions. For example, itaconation on KEAP1 blocks the KEAP1-mediated degradation of Nrf2, allowing the translocation of Nrf2 into the nucleus and the induction of antioxidant genes expression.^6^ Up to now, more crucial itaconation target proteins have been identified, which are widely involved in immune regulation,^9,10^ antioxidant response,^6^ cell death^11–13^ and glucose metabolism.^14,15^ However, the identification of itaconation target proteins in viral infection remains limited.

Rab GTPases (Rabs) is the largest family of small GTPases, serving as multifaceted organizers of almost all membrane trafficking processes, including virus traffic in cells.^16^ The cycling of Rab GTPases between the membrane and cytoplasm is precisely regulated by various proteins.^17^ Rab GDP dissociation inhibitors (GDIs) bind to membrane-bound Rabs and extract Rabs from the membrane, thereby enabling Rabs’ availability for subsequent round of membrane delivery.^18^ The distribution of Rab is believed to have an impact on viral infection; however, this field has not been sufficiently researched.

In this study, we show that IRG1-itaconate axis is activated by viral infection and sequentially promote viral entry, and post-entry process. Specifically, itaconate alkylates Rab GDP dissociation inhibitor beta (GDI2), thereby inhibiting its ability to extract Rabs from the membrane. The maintenance of Rabs on the membrane facilitates the Rab-dependent viral infection. These findings identify the IRG1-itaconate axis as a regulator of viral infection.

## Results

### Inducible itaconate facilitates viral infection

**Fig. 1:**
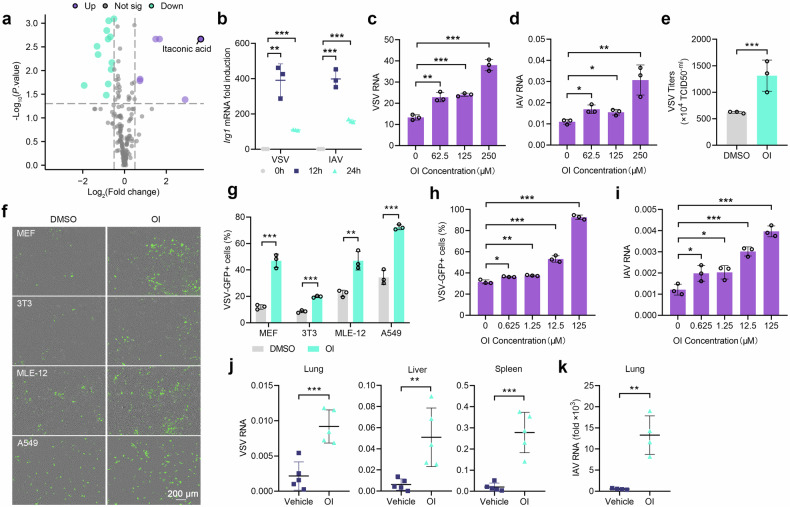
Inducible IRG1-itaconate axis facilitates viral infection. **a** Metabolites in the lungs from mice i.p. infected with VSV or not (*n* = 6). **b** *Irg1* mRNA expression in the lungs of mice intranasally (i.n.) infected with VSV and IAV (*n* = 3). **c**, **d** Viral RNA in PMs pretreated with different concentrations of OI, infected with VSV (**c**) or IAV (**d**) (*n* = 3). **e** Viral titers in the supernatant of PMs pretreated with OI (250 μM) or DMSO (*n* = 3). **f**, **g** VSV-GFP infection in MEF, 3T3, MLE-12 and A549 cells pretreated with OI (125 μM) or DMSO, detected by fluorescence (**f**) and flow cytometry (**g**) (*n* = 3). **h**, **i** VSV-GFP (**h**) and IAV (**i**) infection in MLE-12 cells pretreated with different concentrations of OI. **j**, **k** Mice were pretreated with OI, subsequently infected with VSV (i.p.) and IAV (i.n.), and viral RNA loads were detected (*n* = 5 or 4). Data are mean ± SD or representative of 3 independent experiments with similar results. *p < 0.05, **p < 0.01, ***p < 0.001 by an unpaired, two-tailed *t*-test

To investigate the metabolic alterations following viral infection, metabolomics analysis was performed in mouse lung 12 h after intraperitoneal (i.p.) infection with vesicular stomatitis virus (VSV). The results revealed itaconate as the predominant elevated metabolite post infection (Fig. 1a). Meanwhile, the expression of itaconate synthetase IRG1 in lung, liver, and spleen was significantly increased at 12 h after i.p. infection with VSV or herpes simplex virus type I (HSV-1) (Supplementary Fig. 1a, b). Additionally, nasal infection with VSV and influenza A virus PR8 (IAV) also led to a significant induction of *Irg1* expression at 12 h (Fig. 1b).

Next, we investigated the biological function of itaconate in viral infection. We employed a cell-permeable derivative of itaconate, 4-octyl itaconate (OI), which closely mimics the biological functions of itaconate.^19^ Treatment of peritoneal macrophages (PMs) with OI increased intracellular VSV, IAV (Fig. 1c, d) and HSV-1 (Supplementary Fig. 1c) genome load. Correspondingly, viral titers were confirmed to be higher in culture medium with OI treatment (Fig. 1e and Supplementary Fig. 1d). Similar results were observed in PMs treated with underivatized itaconate as well (Supplementary Fig. 1e–g). Besides macrophages, viruses also infect epithelial cells, endothelial cells and fibroblast cells.^20^ Consistently, OI also enhanced the infection in pulmonary epithelial and fibroblast cell lines including MEF, 3T3, MLE-12 and A549 cells (Fig. 1f, g). Additionally, an OI concentration as low as 0.625 μM was sufficient to promote viral replication in MLE-12 cells (Fig. h, i). We further investigated the in vivo effect of itaconate, and pre-treatment with OI significantly increased their susceptibility to VSV and IAV infections (Fig. 1j, k). These findings indicate that itaconate and OI could promote various viral infections.

Itaconate and its derivatives have been reported to regulate type I interferon (IFN-I) production.^6,19^ To determine whether the effect of itaconate on viral infection was attributed to IFN-I pathway, we explored the effect of itaconate in *Irf3*^-/-^ and *Ifnar1*^*-*/-^ macrophages. Despite the deficiency of IFN-I pathway in these cells, OI still significantly augmented VSV and IAV infection (Supplementary Fig. 2a-e). Similarly, blockade of interferon receptor with blocking antibody failed to abrogate the enhancing effect of OI on VSV and IAV infection (Supplementary Fig. 2f, g). Conversely, OI failed to promote HSV-1 infection in macrophages deficient in IFN-I pathway, indicating its reliance on IFN-I (Supplementary Fig. 2h, i**)**. This is consistent with the previous findings that itaconate exert inhibitory effects on IFN-I production through itaconation of STING.^21,22^ Taken together, these data indicate that OI control VSV and IAV infection independent of IFN-I signaling.

### IRG1 facilitates VSV and IAV infection through itaconate

**Fig. 2:**
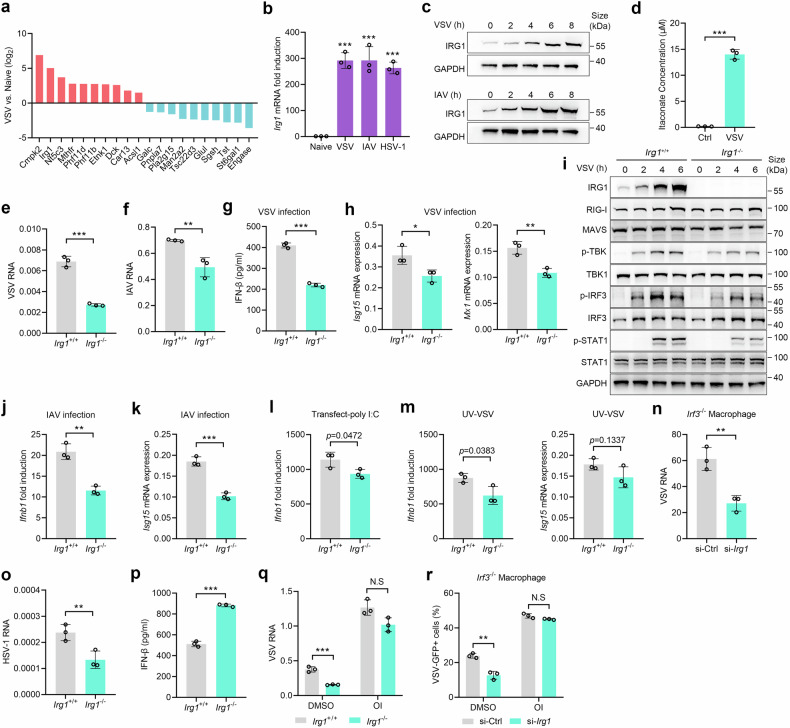
IRG1 facilitates VSV and IAV infection independent of IFN-I pathway. **a** The most significantly altered metabolic genes upon VSV infection were identified by RNA-seq (*n* = 2). **b** *Irg1* mRNA expression in PMs infected with VSV, IAV or HSV-1 for 8 h (*n* = 3). **c** Immunoblot of IRG1 in PMs infected with VSV or IAV. **d** Itaconate in the supernatants of PMs infected with VSV for 12 h (*n* = 3). **e**, **f** Viral RNA in *Irg1*^+/+^ and *Irg1*^-/-^ BMDMs infected with VSV (**e**) or IAV (**f**) (*n* = 3). **g** ELISA analysis of IFN-β in the supernatants of *Irg1*^+/+^ and *Irg1*^-/-^ BMDMs infected with VSV for 24 h (*n* = 3). **h** *Isg15* and *Mx1* mRNA expression in *Irg1*^+/+^ and *Irg1*^-/-^ BMDMs infected with VSV (*n* = 3). **i** Immunoblot of IRG1, RIG-I, MAVS, TBK1, phosphorylated TBK1 (p-TBK1), IRF3, phosphorylated IRF3 (p-IRF3), STAT1, phosphorylated STAT1 (p-STAT1) in *Irg1*^+/+^ and *Irg1*^-/-^ BMDMs infected with VSV. **j**, **k** *Ifnb1* (**j**) and *Isg15* (**k**) mRNA expression in *Irg1*^+/+^ and *Irg1*^-/-^ BMDMs infected with IAV (*n* = 3). **l** *Ifnb1* mRNA expression in *Irg1*^+/+^ and *Irg1*^-/-^ BMDMs transfected with poly I:C (3 ug/ml) for 8 h (*n* = 3). **m** *Ifnb1* and *Isg15* mRNA expression in *Irg1*^+/+^ and *Irg1*^-/-^ BMDMs infected with UV-VSV (*n* = 3). **n** VSV RNA in *Irf3*^-/-^ PMs carrying *Irg1* or control siRNA (*n* = 3). **o** HSV-1 RNA in *Irg1*^+/+^ and *Irg1*^-/-^ BMDMs (*n* = 3). **p** ELISA analysis of IFN-β in the supernatants of *Irg1*^+/+^ and *Irg1*^*-/-*^ BMDMs infected with HSV-1 for 24 h (*n* = 3). **q** VSV RNA in *Irg1*^+/+^ and *Irg1*^-/-^ BMDMs pretreated with OI (250 μM) or DMSO (*n* = 3). **r** The effect of OI on VSV-GFP infection rate in *Irf3*^-/-^ PMs carrying *Irg1* or control siRNA (*n* = 3). Data are mean ± SD or representative of 3 independent experiments with similar results. *p < 0.05, **p < 0.01, ***p < 0.001, N.S, not significant by an unpaired, two-tailed *t*-test

We subsequently investigate the impact of endogenous IRG1 on viral infection. *Irg1* exhibited significant upregulation among metabolic genes upon viral infection (Fig. 2a, b). Viral infection induced significant IRG1 expression (Fig. 2c) and itaconate production (Fig. 2d) in macrophages. Functionally, *Irg1* deficiency attenuated VSV and IAV infection (Fig. 2e, f), resulting in a decreased IFN-I production, ISGs expression, including ubiquitin-like protein ISG15 (*Isg15*) and interferon-induced GTP-binding protein Mx1 (*Mx1*), and TBK1-IRF3 signaling pathway activation (Fig. 2g–k). To further elucidate the role of IRG1 in the immune response to cytoplasmic RNA, we stimulated macrophages with ultraviolet irradiated VSV (UV-VSV) and intracellular transfection of poly I:C. We observed that knockout of IRG1 slightly attenuated the immune responses to UV-VSV and poly I:C (Fig. 2l, m). However, viral infection was paradoxically inhibited in *Irg1*^-/-^ macrophages (Fig. 2e, f), indicating the enhancing effect of IRG1 on RNA virus replication surpasses its impact on innate immune response. Moreover, *Irg1* silencing still impeded VSV infection in *Irf3*^-/-^ macrophages (Fig. 2n and Supplementary Fig. 2j, k). Taken together, these data indicate that endogenous IRG1 improves the susceptibility of macrophages to VSV and IAV infection through an IFN-I independent manner. In contrast, *Irg1* loss reduced HSV-1 infection while increased the production of IFN-I (Fig. 2o, p), which can be attributed to its negative regulation of the STING-mediated immune response.^23^

Considering that endogenous IRG1 can function in an itaconate-independent manner,^24,25^ we treated *Irg1*^-/-^ macrophages with OI following viral infection. The supplementation of OI to *Irg1*^-/-^ macrophages greatly enhanced VSV infection and rescued the impaired viral infection caused by *Irg1* deficiency (Fig. 2q), even in an IFN-I-deficient situation (Fig. 2r). These data suggest that the IRG1-itaconate axis promotes viral infection independent of IFN-I.

### Antioxidant alterations is not responsible for OI-mediated facilitation of viral infection

We next investigated the mechanisms underlying the promotion of VSV and IAV infection by itaconate. The biological functions of the IRG1-itaconate axis are suggested to rely on its regulation of antioxidant response.^26^ Indeed, OI suppressed ROS production in macrophages with VSV infection (Supplementary Fig. 3a, b). However, ROS scavengers, NAC (N-acetyl-L-cysteine) and Tempol, had no effect on OI-mediated enhancement of VSV infection (Supplementary Fig. 3c, d). Furthermore, Nrf2 is characterized as a sensor of oxidative stress and regulated by itaconate.^6^ Consistently, we found that OI significantly increased Nrf2 translocation into the nucleus upon VSV infection (Supplementary Fig. 3e). However, *Nrf2* silencing did not affect OI-induced viral overgrowth (Supplementary Fig. 3f–h). Thus, the promotion of VSV infection by OI is not mediated through the regulation of antioxidant responses.

### Glucose metabolism disturbance is not responsible for OI-mediated facilitation of viral infection

Itaconate also exerts a regulatory effect on glycolysis and oxidative respiration by specifically targeting key enzymes such as GAPDH^14,15^ and SDH.^27^ Therefore, we investigated the role of glucose metabolism in facilitating viral infection via OI. ATP significantly enhanced viral replication (Supplementary Fig. 4a). However, the activation of IRG1-itaconate axis reduced both ECAR and OCR (Supplementary Fig. 4b, c), suggesting the negative regulation of glycolysis, oxidative respiration, and impaired ATP generation. Furthermore, ATP synthase inhibitor, oligomycin, significantly impeded viral replication, while the enhancing effect of OI on viral infection remained unaffected (Supplementary Fig. 4d).

A recent report revealed that neuronal itaconate inhibits the activity of SDH, leading to a metabolic state that suppresses Zika virus replication.^27^ To determine this in our situation, we pretreated macrophages with dimethyl malonate (DMM), a classical inhibitor of SDH, and found that DMM had no significant effect on VSV infection in vitro (Supplementary Fig. 4e-g). Furthermore, in vivo data revealed that neither viral burden nor cytokines production was affected in mice treated with DMM (Supplementary Fig. 4h, i). Taken together, these findings indicate that the regulation of glucose metabolism via the IRG1-itaconate axis is not a pivotal factor in promoting VSV infection.

### Geranylgeranyl diphosphate is required for OI-mediated facilitation of viral infection

**Fig. 3:**
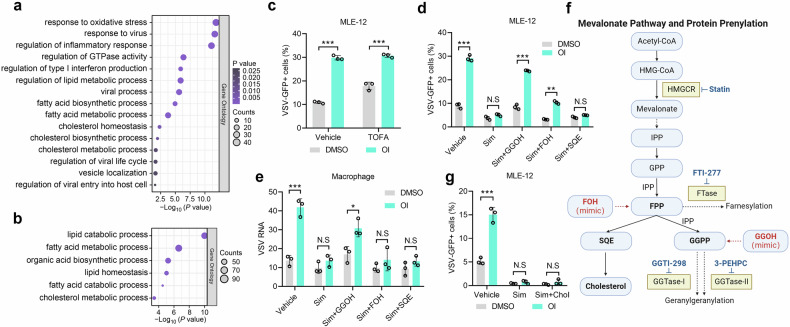
GGPP is indispensable for facilitating viral infection induced by OI. **a**, **b** GO Pathway enrichment analysis of differentially expressed genes from the RNA-seq data of *Irg1*^+/+^ and *Irg1*^-/-^ BMDMs (**a**), and PMs treated with OI (250 μM) or DMSO (**b**). **c** The effect of OI on VSV-GFP infection rate in MLE-12 cells pretreated with TOFA (30 μM) or vehicle (*n* = 3). **d**, **e** The effect of OI on VSV infection in MLE-12 cells (**d**) and PMs (**e**) pretreated with simvastatin (Sim, 10 μM) plus GGOH (15 μM), FOH (15 μM), SQE (15 μM) or not (*n* = 3). **f** Schematic of mevalonate pathway and protein prenylation. **g** The effect of OI on VSV-GFP infection rate in MLE-12 cells pretreated with simvastatin (Sim, 10 μM) with or without a cell permeable cholesterol (Chol, 5 ug/ml) (*n* = 3). Data are mean ± SD. *p < 0.05, **p < 0.01, ***p < 0.001, N.S, not significant by an unpaired, two-tailed *t*-test

To identify the mechanisms of the IRG1-itaconate activity during viral infection, we performed transcriptome RNA-sequencing analysis in *Irg1*^+/+^ and *Irg1*^-/-^ macrophages. Differentially expressed genes are significantly enriched in the pathways related to the classical functions of itaconate, such as “response to oxidative stress” and “regulation of inflammatory response” (Fig. 3a). Additionally, we observed a significant enrichment of differentially expressed genes in the pathways associated with fatty acid metabolism and cholesterol metabolism (Fig. 3a), suggesting the potential involvement of the IRG1-itaconate axis in lipid metabolism. Consistently, OI treatment also induced a broad change in genes related to lipid metabolism-associated signaling pathways, especially cholesterol and fatty acid metabolic processes (Fig. 3b). Therefore, we next focused on the relationship between the IRG1-itaconate axis and lipid metabolism.

Firstly, we investigated the role of fatty acids and cholesterol synthesis in OI-mediated viral overgrowth. TOFA, an inhibitor of fatty acids synthesis, did not affect viral overgrowth caused by OI (Fig. 3c and Supplementary Fig. 5a). However, simvastatin and lovastatin, inhibitors of the rate-limiting enzyme HMG-CoA reductase (HMGCR) in cholesterol synthesis,^28^ strongly abrogated the viral overgrowth caused by OI (Fig. 3d, e and Supplementary Fig. 5b), indicating that OI-mediated viral overgrowth largely depends on some metabolic pathways downstream of HMGCR.

Inhibition of HMGCR is known to reduce the metabolic intermediate mevalonate, which ultimately results in a drop in cholesterol and other biosynthetic intermediate isoprenoids, including farnesyl diphosphate (FPP) and geranylgeranyl diphosphate (GGPP)^29^ (Fig. 3f). To determine the key intermediates, we analyzed whether OI affected cholesterol production and distribution. Neither *Irg1* deficiency nor OI treatment changed total- or free-cholesterol levels in MLE-12 cells and macrophages (Supplementary Fig. 5c, d). The distribution of cholesterol remained unaffected upon treatment with OI (Supplementary Fig. 5e). In addition, supplementation of squalene (SQE) (Fig. 3d, e), the precursor of cholesterol, or a cell permeable form of cholesterol (Fig. 3g and Supplementary Fig. 5f) failed to rescue the activity of statins on OI-mediated viral overgrowth, indicating that the effect of OI is independent of cholesterol.

We next investigated the role of isoprenoids on viral infection induced by OI. In mevalonate pathway, mevalonate sequentially transforms to isopentenyl pyrophosphate (IPP), FPP and GGPP (Fig. 3f). Addition of permeable geranylgeraniol (GGOH), the donor of a geranylgeranyl group (Fig. 3f), almost fully rescued the inhibitory activity of simvastatin in both MLE-12 cells and macrophages (Fig. 3d, e). Conversely, farnesol (FOH), which acts as a donor of a farnesyl group and was unable to rescue GGPP synthesis in the absence of IPP^28^ (Fig. 3f), exhibited an inability to restore the observed OI effect in macrophages (Fig. 3e). Collectively, these data suggest that isoprenoids, especially GGPP, may be implicated in the impact of OI on viral infection.

### Itaconate promotes the membrane localization of Rab GTPases

GGPP serves as the substrate for a distinct type of protein prenylation called geranylgeranylation (Fig. 3f). Protein prenylation is responsible for anchoring soluble proteins to cellular membranes.^29^ Geranylgeranylation is primarily catalyzed by two heterodimeric prenyltransferases, namely geranylgeranyltransferase type I (GGTase-I), and Rab geranylgeranyl transferase (GGTase-II, RGGT, RabGGTase)^30^ (Fig. 3f). These two prenyltransferases exhibit specificity towards certain target proteins.

**Fig. 4:**
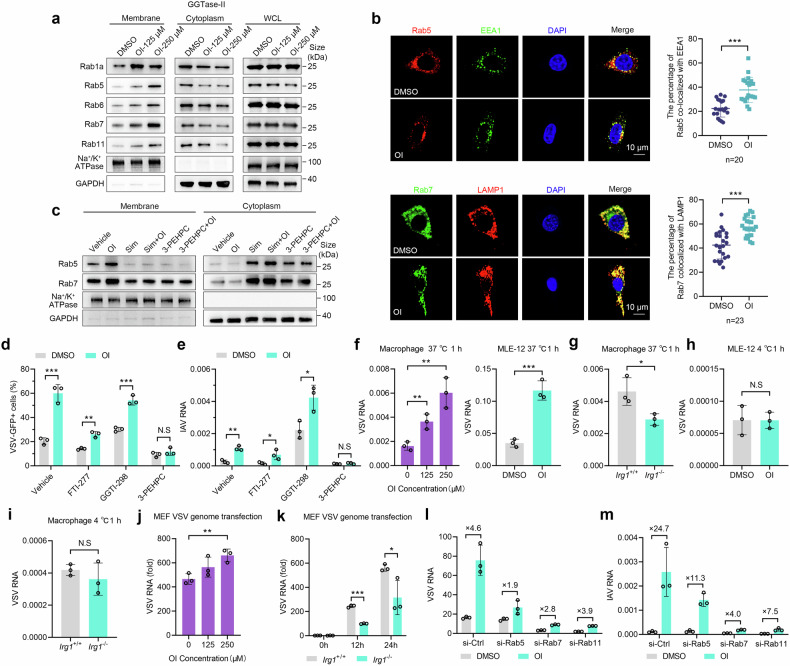
OI induces the redistribution of Rab GTPases to enhance viral infection. **a** Immunoblot of GGTase-II substrate proteins in the membrane, cytoplasm and total cell fractions of MLE-12 cells pretreated with OI (125 μM) or DMSO. **b** Confocal of Rab5 and EEA1 co-localization (*n* = 20), Rab7 and LAMP1 co-localization (*n* = 23), in MLE-12 cells pretreated with OI or DMSO. The icon on the right displays the co-localization rate. **c** The effect of OI on Rab5 and Rab7 distribution in the membrane and cytoplasm in MLE-12 cells treated with simvastatin (Sim, 10 μM), 3-PEHPC (1.5 mM) or vehicle as indicated. **d**, **e** VSV-GFP (**d**) and IAV (**e**) infection in MLE-12 cells pretreated with OI or DMSO plus FTI-277 (10 μM), GGTI-298 (10 μM), 3-PEHPC (1.5 mM) or vehicle for 12 h (*n* = 3). **f** VSV RNA in PMs and MLE-12 cells pretreated with OI or DMSO 1 h post infection (*n* = 3). **g** VSV RNA in *Irg1*^+/+^ and *Irg1*^-/-^ BMDMs infected with VSV for 1 h (*n* = 3). **h**, **i** VSV RNA in MLE-12 cells pretreated with OI or DMSO (**h**), and in *Irg1*^+/+^ and *Irg1*^-/-^ BMDMs (**i**) infected with VSV for 1 h at 4 °C (*n* = 3). **j**, **k** VSV RNA in MEF cells pretreated with OI or DMSO (**j**), and in *Irg1*^+/+^ and *Irg1*^-/-^ MEF cells (**k**) transfected with VSV-RNA (*n* = 3). **l**, **m** The effect of OI on VSV-GFP (**l**) and IAV (**m**) infection in MLE-12 cells silenced of Rab5, Rab7, or Rab11 (*n* = 3). Data are mean ± SD or representative of 3 independent experiments with similar results. *p < 0.05, **p < 0.01, ***p < 0.001, N.S, not significant by an unpaired, two-tailed *t*-test

To investigate whether OI affected the subcellular localization of the classical target proteins of GGTase-I and GGTase-II, the cytoplasmic and membrane proteins of MLE-12 cells treated with OI or vehicle were isolated, followed by immunoblot analysis. The result displayed that OI had no effect on the distribution of GGTase-I’s target proteins, including Rac, Rap and RhoA (Supplementary Fig. 6a). However, it significantly increased the membrane localization of Rabs (including Rab1a, Rab5, Rab6, Rab7 and Rab11), the classical targets of GGTase-II, and decreased their cytoplasmic localization without altering total protein levels (Fig. 4a). Rab5 and Rab7, which localized on early endosomes and late endosomes respectively, exhibited a greater tendency towards endosomal membrane localization upon OI treatment (Fig. 4b). Moreover, the regulatory impact of OI on Rabs’ distribution was abolished by 3-PEHPC, a GGTase-II inhibitor, or simvastatin that blocks GGPP production (Fig. 4c). These data suggest that OI enhances the subcellular distribution of Rabs on the membrane, while GGPP and GGTase-II-mediated geranylgeranylation on Rabs is necessary for this effect. Additionally, the promotion of VSV and IAV infection by OI was abolished by GGTase-II inhibitor 3-PEHPC, but not GGTase-I inhibitor GGTI-298 (Fig. 4d, e). A similar result was observed upon itaconate treatment (Supplementary Fig. 6b). Taken together, these data indicate that GGTase-II-mediated geranylgeranylation is required for regulating the localization of Rabs and enhancing viral infection by OI.

In addition, we also investigated another type of protein prenylation known as farnesylation, which is mediated by the FPP-farnesyltransferase (FTase) axis (Fig. 3f). OI induced a redistribution of Ras, a classical target protein of FTase, between cytoplasm and membrane (Supplementary Fig. 6c). However, the enhancing effect of OI on viral infection remained unaffected by treatment with the FTase inhibitor FTI-277 (Fig. 4d, e). Thus, FTase is not responsible for the observed effect of OI on viral infection.

### The IRG1-itaconate axis promotes viral entry and post-entry processes

Given that viruses exploit Rab GTPases, the master regulators of intracellular membrane trafficking, to facilitate their entry, uncoating and budding processes,^16^ we explored the specific life stages in which the IRG1-itaconate axis affects viral infection. Notably, OI increased VSV entry in a dose-dependent manner (Fig. 4f), while *Irg1* deficiency impaired VSV entry in macrophages (Fig. 4g). Meanwhile, neither OI treatment nor *Irg1* deficiency affected viral binding (Fig. 4h, i). Thus, the IRG1-itaconate axis acts on the early entry of VSV infection.

To assess the impact of the IRG1-itaconate axis on post-entry processes of viral infection, the cells were transfected with viral genomic RNA to bypass the binding and entry stages of VSV infection. VSV genome load was also elevated by OI (Fig. 4j). In accordance, viral genome load was significantly lower in *Irg1*^-/-^ group (Fig. 4k). Collectively, these results reveal that the IRG1-itacoante axis promotes VSV infection via facilitating both viral entry and post-entry processes.

We next investigated the role of the crucial Rab GTPases associated with viral entry, uncoating and egress processes.^16,31^ Rab5, Rab7 or Rab11was silenced respectively following viral infection (Supplementary Fig. 7a-c). Indeed, silencing Rab5, Rab7 and Rab11 attenuated the increased VSV and IAV infections induced by OI (Fig. 4l, m). Therefore, the impact of OI on viral infection is dependent on various Rabs associated with viral entry and post-entry processes.

### Itaconation of GDI2 impedes the extraction of Rab GTPases from the membrane

**Fig. 5:**
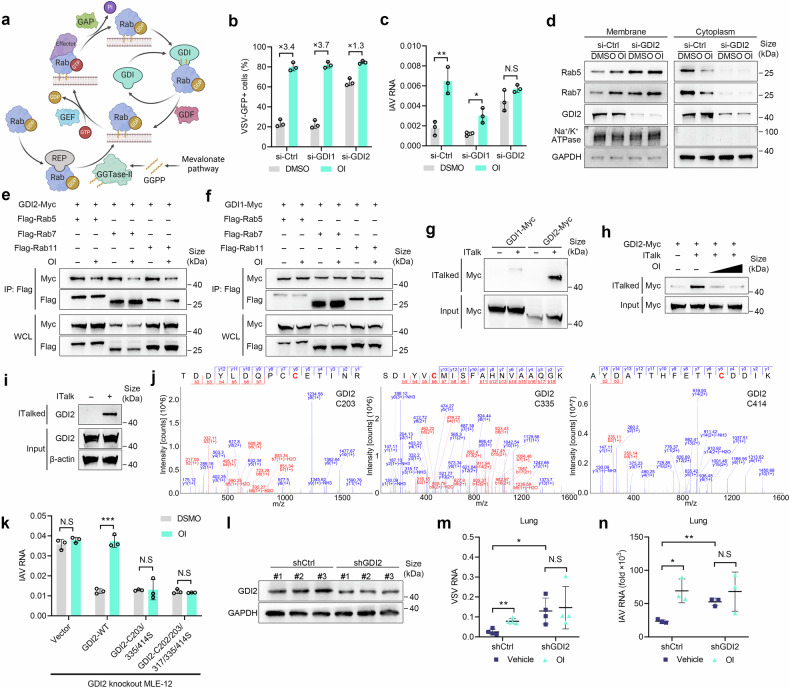
OI inhibits the extraction of Rabs from the membrane via alkylation of GDI2. **a** Schematic of Rab GTPases cycle. **b**, **c** The effect of OI on VSV-GFP (**b**) and IAV (**c**) infection in MLE-12 cells silenced of GDI1 or GDI2 (*n* = 3). **d** Immunoblot of Rab5 and Rab7 in the membrane and cytoplasm of MLE-12 cells carrying GDI2 or control siRNA, and treated with OI (125 μM) or DMSO. **e**, **f** The effect of OI on the interaction of GDI2 (**e**) and GDI1 (**f**) with the Rabs in HEK293T cells transfected with GDI2-Myc or GDI1-Myc together with Flag-Rab5, Flag-Rab7 or Flag-Rab11. **g** Immunoblot of ITalk-modified (ITalked) GDI1 and GDI2 in HEK293T cells transfected with GDI1-Myc or GDI2-Myc. **h** Immunoblot of ITalked GDI2 in HEK293T cells treated with different concentrations of OI (0, 62.5 or 125 μM). **i** ITalked endogenous GDI2 proteins in MLE-12 cells treated with ITalk or DMSO. **j** LC-MS/MS analysis of itaconation on GDI2 in HEK293T cells transfected with GDI2-Flag, treated with OI for 12 h. **k** The effect of OI on IAV infection in GDI2-deficient MLE-12 cells transfected with wildtype or mutated GDI2 (*n* = 3). **l** Immunoblot of GDI2 in the lungs from mice intratracheally administered with adeno-associated virus containing GDI2 shRNA (shGDI2) or scrambled shRNA (shCtrl) (*n* = 3 per group). **m** VSV RNA in the lungs of mice upon GDI2 knockdown and control mice (*n* = 4). **n** IAV RNA in the lungs of mice upon GDI2 knockdown and control mice (*n* = 3). Data are mean ± SD or representative of 3 independent experiments with similar results. *p < 0.05, **p < 0.01, ***p < 0.001, N.S, not significant by an unpaired, two-tailed *t*-test

Subsequently, we aim to elucidate the mechanism by which itaconate regulates the subcellular localization of Rab proteins. Once a Rab protein is synthesized, it is escorted by Rab escort protein (REP) to GGTase-II for geranylgeranylation, thereby acquiring the ability to bind to membranes. Subsequently, Rabs shuttle between the membrane and cytoplasm in a tightly regulated manner regulated by various proteins^17^ (Fig. 5a). We initially investigated whether OI affected the geranylgeranylation on Rab proteins using GGOH-azide, a probe for studying geranylgeranylation.^32,33^ OI treatment resulted in a decrease of the geranylgeranylation of Rabs, either overall or individually (Supplementary Fig. 8a–c**)**, indicating that OI does not increase the membrane delivery of Rabs through up-regulating the geranylgeranylation of Rabs.

Increased membrane localization of Rabs can be attributed to an increased delivery to membrane or a reduced dissociation from it, we subsequently focused on the dissociation process of Rabs from the membrane. Rab GDP dissociation inhibitors (GDI1 and GDI2) bind to geranylgeranylation groups on the inactive form of Rabs and extract them from the membrane and enabling their availability for another round of membrane delivery^18^ (Fig. 5a). Dysfunction of GDIs leads to the retention of Rabs within the membrane. To determine whether OI-induced viral overgrowth was dependent on GDIs, we respectively silenced GDI1 and GDI2 (Supplementary Fig. 9a, b). Notably, silencing GDI2 but not GDI1 abolished the promotion of VSV and IAV infection caused by OI (Fig. 5b, c). Moreover, silencing GDI2 significantly impeded the dissociation of Rab5 and Rab7 from the membrane, resulting in an elevation of membrane-bound Rabs and a reduction of cytoplasmic Rabs (Fig. 5d). Concurrently, OI-induced redistribution of Rab5 and Rab7 between cytoplasm and membrane was abolished in the absence of GDI2 (Fig. 5d). Therefore, these data indicate that OI inhibits GDI2-mediated extraction of Rabs from the membrane, resulting in enhancement of Rabs’ distribution on the membrane and subsequent viral infection.

Considering that itaconation could hinder protein-protein interactions,^6,11^ we supposed that itaconation on GDI2 might hinder the interaction between GDI2 and Rabs, resulting in a decreased extraction of Rabs from membrane. Indeed, OI attenuated the interaction between Rabs and GDI2, but have no effect on its interaction with GDI1 (Fig. 5e, f). Itaconate-alkyne (ITalk),^34^ a probe for itaconation, was utilized to enrich proteins modified by itaconate. The enrichment of GDI2 was observed in the ITalk-labeled proteins (Fig. 5g) from 293 T cells transfected with GDI2-Myc, which could be attenuated by the addition of OI (Fig. 5h), suggesting that both ITalk and OI could alkylate GDI2 and target the same sites. Furthermore, the enrichment of endogenous GDI2 was also observed in the ITalk-labeled proteins from MLE-12 cells (Fig. 5i). The sites of itaconation on GDI2 were subsequently identified, revealing the presence of five alkylated sites (C202, C203, C317, C335, C414), among which three sites (C203, C335, C414) exhibited stable detectability across various experiments (Fig. 5j). These data indicate that GDI2 is a direct target for itaconation.

To identified the critical itaconation sites on GDI2 that contributed to the enhancement of viral infection induced by OI, GDI2-knockout cells were transfected with wildtype GDI2, mutant GDI2 or an empty vector. In the absence of GDI2, OI-induced viral overgrowth was completely abolished (Fig. 5k). Transfection of both wildtype and mutated GDI2 suppressed viral infection; however, only wildtype GDI2 reinstated the enhancing effect of OI on viral infection (Fig. 5k). The C203/C335/C414 mutation of GDI2 was found to be sufficient for blocking the effect of OI (Fig. 5k), demonstrating the indispensability of these three sites within GDI2. The collective data indicate that the addition of itaconate lead to the itaconation of C203/335/414 residue on GDI2, thereby disrupting the interaction between GDI2 and Rabs, increasing Rabs’ membrane distribution, and finally promoting viral overgrowth.

Finally, mice were intranasally administered with adeno-associated virus (AAV) containing GDI2 shRNA to establish a knockdown model of GDI2 (Fig. 5l). Knocking down GDI2 promoted viral infection in lung tissues (Fig. 5m, n), while also abolished the promotion of VSV and IAV infection caused by OI treatment (Fig. 5m, n). These findings indicate that GDI2 inhibits VSV and IAV infection, whereas OI facilitates viral infection through its modification of GDI2 in vivo.

### Viral infection induces robust IRG1-itaconate axis activation in neutrophils

**Fig. 6:**
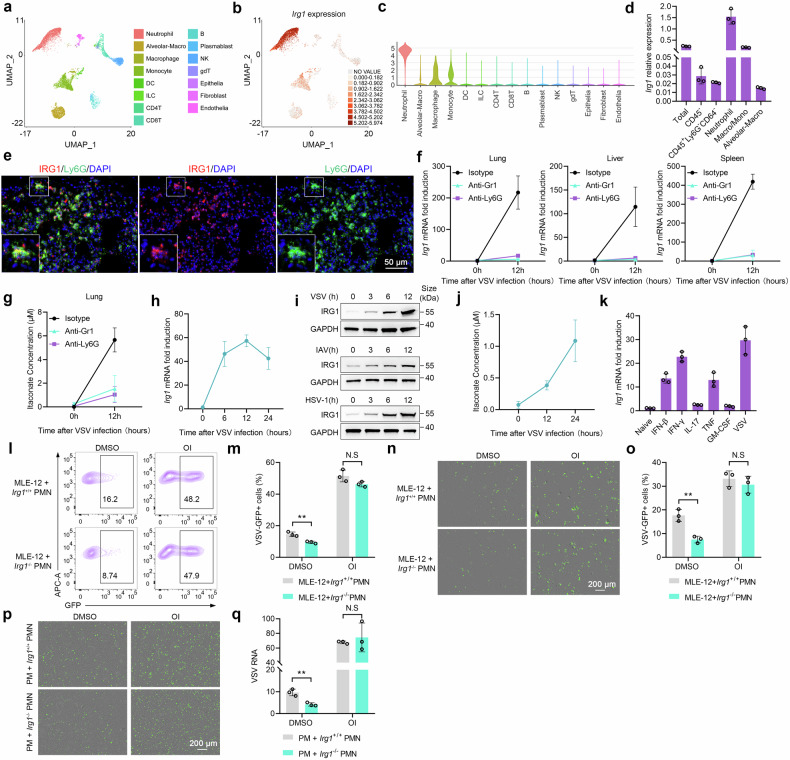
Viral infection induces robust IRG1-itaconate axis activation in neutrophils. **a**, **b** UMAP clusters (**a**) and *Irg1* expression (**b**) of scRNA-seq data obtained from the lung of mouse i.p. infected with VSV for 12 h. **c** Violin plots showing *Irg1* expression in each cluster. **d** *Irg1* mRNA expression in different cells sorted from the VSV infected lungs (*n* = 3). **e** Immunofluorescence analysis of IRG1 and Ly6G expression in VSV infected lungs. **f**, **g** *Irg1* mRNA expression in the lungs, livers and spleens (**f**), and itaconate accumulation in the lung (**g**) of neutrophil-depleted and control mice (*n* = 3). **h** *Irg1* mRNA expression in neutrophils infected with VSV (*n* = 3). **i** Immunoblot of IRG1 in neutrophils infected with VSV, IAV or HSV-1. **j** Itaconate in the supernatant of neutrophils infected with VSV (*n* = 3). **k** *Irg1* mRNA expression in neutrophils stimulated with IFN-β (100 ng/ml), IFN-γ (100 ng/ml), IL-17 (100 ng/ml), TNF (100 ng/ml), GM-CSF (100 ng/ml) or VSV for 8 h (*n* = 3). **l**–**o** VSV-GFP infection rate in MLE-12 cells co-cultured with *Irg1*^+/+^ or *Irg1*^-/-^ neutrophils (ratio = 1:1), pretreated with OI or DMSO (**l**, **m**) (*n* = 3). A transwell system was used in (**n**, **o**) (*n* = 3). **p**, **q** VSV-GFP infection in PMs co-cultured with *Irg1*^+/+^ or *Irg1*^-/-^ neutrophils as in (**l**), was revealed through fluorescence (**p**), qRT-PCR (**q**) (*n* = 3). Data are mean ± SD or representative of 3 independent experiments with similar results. **p < 0.01, N.S, not significant by an unpaired, two-tailed *t*-test

Neutrophils-derived itaconate attracts increasing attention in recent years, playing a pivotal role in cancer,^13,35^ trauma^36^ and bacterial infection.^37^ We next identified the cell types responsible for itaconate production during viral infection by single-cell RNA sequencing (scRNA-seq). Observably, neutrophils possessed the highest *Irg1* expression (Fig. 6a–c) upon viral infection. Macrophages and monocytes also exhibited moderate *Irg1* expression, albeit to a significantly lesser extent than neutrophils (Fig. 6c). Among macrophages, interstitial macrophages exhibited certain level of IRG1 expression, whereas alveolar macrophages demonstrated minimal expression (Fig. 6c). These data were further confirmed by sorting these cell subpopulations post infection (Fig. 6d and Supplementary Fig. 10a). Furthermore, immunofluorescence analysis showed a widespread co-staining of IRG1 with neutrophils (Fig. 6e) and a slight co-staining with macrophages in the lung (Supplementary Fig. 10b).

To further confirm the predominant contribution of neutrophils as the source of itaconate during viral infection, we assessed *Irg1* expression and itaconate production in the organs from neutrophil-depleted mice (Supplementary Fig. 10c). The neutrophil-depleted mice exhibited significantly impaired expression of *Irg1* and itaconate production (Fig. 6f, g). In vitro analysis also confirmed that viral infection induced *Irg1* expression and itaconate production in neutrophils (Fig. 6h–j). In addition, cytokines may also play a crucial role in inducing IRG1 expression in neutrophils (Fig. 6k). Taken together, these data indicate that viral infection induces robust IRG1-itaconate axis activation in neutrophils, which may exert regulatory effects on the progression of viral infection.

To assess the impact of neutrophil-derived itaconate on viral infection, we established a co-culture system comprising pulmonary epithelial MLE-12 cells with *Irg1*^+/+^ or *Irg1*^-/-^ neutrophils. VSV preferred to infect epithelial cells instead of neutrophils (Supplementary Fig. 10d). The co-culture with *Irg1*^+/+^ neutrophils resulted in an augmented susceptibility of MLE-12 to VSV infection compared to the *Irg1*^-/-^ group (Fig. 6l, m). Moreover, supplementation of OI further enhanced viral infection and abrogated the disparities caused by the difference of neutrophils (Fig. 6l, m). To determine whether this effect is dependent on cell contact between neutrophils and epithelial cells, a transwell system was employed. In the situation, *Irg1*^+/+^ neutrophils still enhanced the susceptibility of MLE-12 cells to VSV infection (Fig. 6n, o), indicating that the secretion of itaconate played a significant role. Similar results were generated when *Irg1*^+/+^ or *Irg1*^*-/-*^ neutrophils were co-cultured with macrophages (Fig. 6p, q). Taken together, the in vitro data suggested that neutrophils secrete an abundant amount of itaconate, thereby creating an environment that facilitates viral infection in epithelial cells and macrophages. Although the impact of macrophage-derived itaconate on their susceptibility to viruses was notable (Fig. 2), the exogenous high concentration of neutrophils-derived itaconate could play a more predominant role in viral infection.

### IRG1-itaconate axis in neutrophils controls viral infection in vivo

**Fig. 7:**
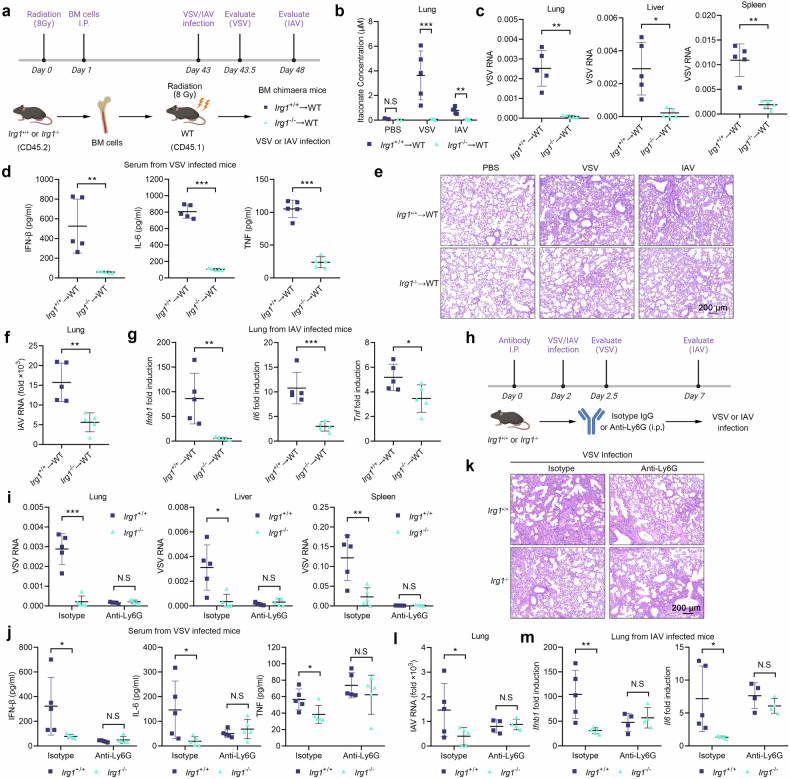
Neutrophils-derived itaconate facilitates VSV and IAV infection. **a** The model of chimeric mice experiments. **b** Itaconate in the lungs of chimeric mice infected with VSV or IAV (*n* = 2 or 5). **c** VSV RNA in the lungs, spleens and livers of chimeric mice (*n* = 5). **d** ELISA analysis of IFN-β, IL-6 and TNF-α in the serum of mice from (**c**) (*n* = 5). **e** H&E of lung sections of mice from (**b**). **f** IAV RNA in the lungs of chimeric mice (*n* = 5). **g** *Ifnb1*, *Il6* and *Tnf* mRNA expression in the lungs of mice from (**f**) (*n* = 5). **h** The model of neutrophils deleting experiments. **i** VSV RNA in the lungs, spleens and livers of neutrophils-deleted and control mice (*n* = 5). **j** ELISA analysis of IFN-β, IL-6 and TNF-α in the serum of mice from (**i**) (*n* = 5). **k** H&E of lung sections of mice from (**i**). **l** IAV RNA in the lungs of neutrophils-deleted and control mice (*n* = 4 or 5). **m** *Ifnb1* and *Il6* mRNA expression in the lungs of mice from (**l**) (*n* = 4 or 5). Data are mean ± SD or representative of 3 independent experiments with similar results. *p < 0.05, **p < 0.01, ***p < 0.001, N.S, not significant by an unpaired, two-tailed *t*-test

Given that IRG1 was mainly expressed in neutrophils and macrophages during viral infection, and CD45^-^ non-immune cells exhibited minimal expressed IRG1 (Fig. 6d), we next investigated whether the IRG1-itaconate axis regulates viral infection in vivo through immune cells by generating *Irg1* immune lineage-deficient mice (Fig. 7a). Itaconate production was barely detectable in the lungs of *Irg1*^-/-^ chimeric mice upon viral infection (Fig. 7b), confirming that itaconate is mainly synthesized by immune cells. Moreover, VSV genomic RNA in the organs from *Irg1*^-/-^ chimeric mice (*Irg1*^-/-^→WT) were significantly reduced compared to *Irg1*^+/+^ chimeric (*Irg1*^+/+^→WT) mice (Fig. 7c). Meanwhile, *Irg1*^-/-^ chimeric mice produced less cytokines in the serum (Fig. 7d) and exhibited reduced infiltration of inflammatory cells in lungs (Fig. 7e). Similar results were obtained with IAV infection (Fig. 7e–g). The above data demonstrate that IRG1-itaconate axis in immune cells enhances in vivo viral infection and induces stronger immune responses.

To further identify the effect of neutrophil-derived itaconate, we depleted neutrophils with anti-Ly6G antibody in *Irg1*^+/+^ and *Irg1*^-/-^ mice (Fig. 7h). Compared with their littermate control, *Irg1*^-/-^ mice showed significantly reduced viral load after infection, accompanied by reduced cytokine production and pathological injures (Fig. 7i–k). However, depletion of neutrophils abrogated the differences between *Irg1*^+/+^ and *Irg1*^-/-^ mice (Fig. 7i–k). The similar effect was observed during IAV infection (Fig. 7l, m). Therefore, neutrophils contribute mostly to the effect of the IRG1-itaconate axis on viral infection.

**Fig. 8:**
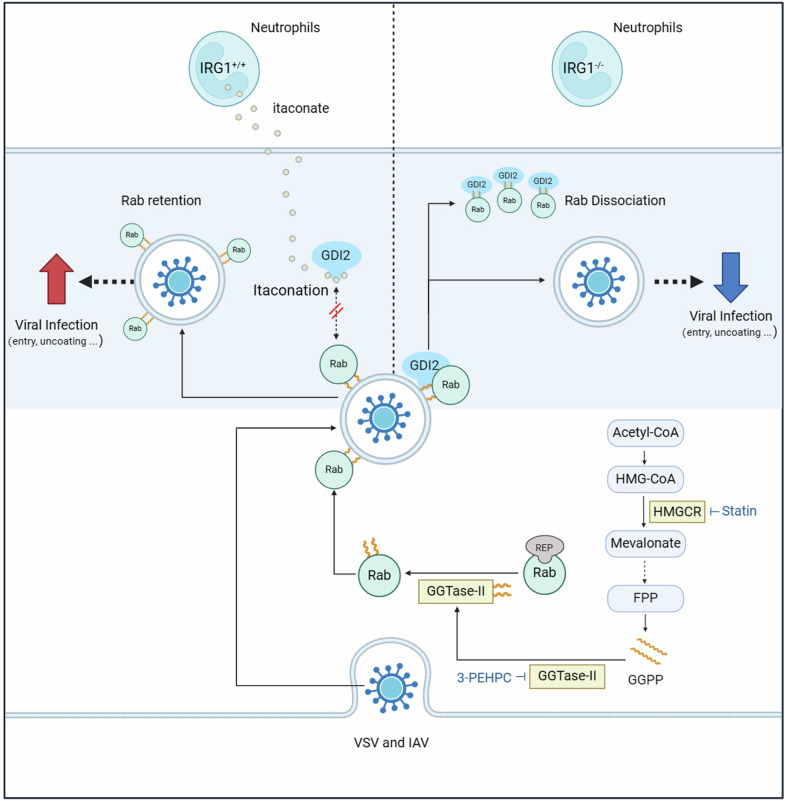
Neutrophils-derived itaconate facilitates viral infection through inducing Rab GTPases redistribution. VSV and IAV infections induce a robust activation of the IRG1-itaconate axis in neutrophils. Subsequently, the secreted itaconate enters macrophages and epithelial cells, where it alkylates GDI2 and impedes its extraction of Rab GTPases from the membrane. As a result, there is an increased retention of Rabs on the membrane, ultimately facilitating Rabs-dependent viral infection

In summary, our study demonstrates that itaconate derived from neutrophils facilitates viral infection by directly alkylating GDI2 and retaining Rab GTPases within the membrane (Fig. 8). This finding enhances our understanding of the IRG1-itaconate axis in viral infection and highlights IRG1 as a potential therapeutic target for certain viral infections.

## Discussion

IRG1-itaconate axis is closely associated with numerous diseases,^25,38^ particularly infectious diseases caused by bacteria and viruses. The antibacterial role of itaconate is widely acknowledged due to its inhibition of bacterial isocitrate lyase (ICL), which blocks the glyoxylate shunt required for optimal growth and pathogenicity.^39^ Itaconate was also shown to be precisely delivered to *Salmonella*-containing vacuoles to limit bacterial growth.^40^ Moreover, itaconate induces TFEB-mediated lysosomal biogenesis to facilitate the clearance of *Salmonella typhimurium*,^41^ and export of itaconate by ABCG2 restricts its antibacterial activity.^42^ Whereas, the latest researches suggest that itaconate may also serve as a facilitator for bacterial infection. Itaconate is exploited by *Staphylococcus aureus*^43^ and *Pseudomonas aeruginosa*^44^ to fuel biofilm formation. In addition, itaconate also impairs survival and bactericidal activity of neutrophils by inhibiting glycolysis and oxidative burst.^37^

Contradictory roles of itaconate in viral infection has also been demonstrated.^26,27,45,46^ It has been reported that itaconate restricts Zika virus replication by inhibiting SDH in neurons.^27^ and restricts SARS-CoV-2 replication by inhibiting Nrf2 activation.^26^ Our study demonstrates the enhancing effect of IRG1-itaconate-axis on VSV and IAV infection independent on the above two antiviral mechanisms. The inhibitory effect of itaconate on IAV infection has been previously observed in A549, PBMC and MDCK cells.^45,47,48^ A549 cells are commonly used for assessing IAV infection, and both itaconate and OI exhibit inhibitory effects on viral titers but not intracellular viral RNA.^47,48^ We also observed that itaconate and OI would not change IAV RNA load in A549 (data not shown), but they can significantly increase IAV RNA load in mouse macrophages and MLE-12 cells. Therefore, we speculate that the effect of itaconate on IAV infection is distinctive in different cell types. Moreover, our in vivo experiments reveal a more convincing effect of itaconate on IAV: in vivo treatment of OI significantly increased IAV infection, while *Irg1*^-/-^ mice showed significantly reduced viral load in lungs after IAV infection, suggesting that the IRG1-itaconate axis is more likely to exert a facilitating role in IAV infection. Moreover, the enhancing effect of itaconate on VSV infection has been observed by two other groups^26,46^; however, the underlying mechanisms have not been elucidated. Therefore, the enhancing effect of itaconate on viral infections is significant as well, but not received adequate attention and research. Our study demonstrates that the IRG1-itaconate axis enhances viral infection, including VSV and IAV, thereby further substantiating the dual role of itaconate in viral infections.

Itaconation is firstly identified on KEAP1, which blocks the KEAP1-mediated degradation of Nrf2 and facilitates the translocation of Nrf2 into the nucleus, thereby inducing antioxidant genes expression.^6^ Similarly, itaconation on TFEB disrupts mTOR/14-3-3 mediated cytosolic retention of TFEB, inducing TFEB nuclear translocation and lysosomal biogenesis.^41,49^ A growing number of itaconation targets have been identified, indicating that the IRG1-itaconate axis is involved in a wide range of biological activities.^34^ Our data demonstrate that OI can alkylate two opposing regulators, GDI2 and GGTase-II, in the Rabs cycle. By alkylating GDI2, OI hinders the extraction of Rabs, resulting in an increase of Rabs’ membrane localization. By alkylating GGTase-II, OI attenuates the geranylgeranylation of newly synthesized Rabs, which should lead to a decrease of Rabs’ membrane localization. In the complex situation where both GDI2 and GGTase-II coexist, the effect of OI on GDI2 is dominant. Therefore, we speculate that although OI decreases the level of geranylgeranylation on Rabs, its impact on Rabs’ localization might be insufficient. However, the potent inhibitors of geranylgeranylation, such as simvastatin and 3-PEHPC, which initially and greatly block Rabs retaining on the membrane, disables OI-induced effect on Rabs’ membrane localization and viral overgrowth. Therefore, the effectiveness of OI-GDI2 axis relies on a threshold of geranylgeranylation to ensure an adequate of Rabs for membrane localization.

On the membrane, only the GTP-bound (active) Rabs can interact with effectors but the GDP-bound (inactive) cannot. The conformation transformation (GTP-bound or GDP-bound) acts as a molecular switch for Rabs. The abnormal maintenance of Rabs in their GDP-bound form (inactive) often results in attenuated viral infection.^50–52^ The cycling of Rabs between cytoplasm and membrane is regulated by various proteins, including GDIs. GDIs are responsible for extraction of GDP-bound Rabs into the cytoplasmic pool, thereby regulating the membrane cycle of Rabs. However, the effect of GDIs on viral infection has not been clearly reported. In our results, the impairment of GDI2 enhances cellular susceptibility to viral infections, indicating that GDI2 functions as an antiviral protein. In accordance, OI, function as an inhibitor of GDI2, enhanced cellular susceptibility to the viruses. The antiviral mechanism of GDI2 is highly valuable and deserves further investigation. Kramer et al. discovered that silencing of GDI2 led to vesicle rearrangement and a significant increase in the number of *Tobacco mosaic virus* (TMV) infection foci observed in *Nicotiana benthamiana*.^53^ Based on this, the antiviral mechanism mediated by GDI2 may involve impeding viral exploitation of the host’s vesicle traffic system.

Our data demonstrate that viral infections induce the activation of IRG1-itaconate axis in neutrophils, thereby facilitating viral infection in epithelial cells and macrophages. Mechanistically, itaconate alkylates GDI2 to hinder its extraction of Rabs from the membrane and retain them on the membrane, ultimately leading to viral overgrowth. Our study reveals a significant role for the IRG1-itaconate axis in regulating viral infection and also provides a potential therapeutic target for certain viral infections.

## Materials and methods

### Mice

*Irg1*^-/-^ C57BL/6 J mice were kindly provided by Dr. Yingke Li (Naval Medical University). *Irf3*^-/-^ mice were kindly provided by Dr. Tadatsugu Taniguchi (University of Tokyo). *Ifnar1*^-/-^ C57BL/6 J mice were from Jackson Laboratory. C57BL/6 J mice were obtained from Joint Ventures Sipper BK Experimental Animals (Shanghai, China). Mice were kept and bred in pathogen-free conditions. All animal experiments were undertaken in accordance with the National Institute of Health Guide for the Care and Use of Laboratory Animals with approval of the Scientific Investigation Board of Naval Medical University, Shanghai.

### Cell culture

Peritoneal macrophages (PMs) were collected from mice 4 days after intraperitoneal injection of thioglycolate. Neutrophils were separated from bone marrow cells of mice using Neutrophil Selection Kit. Bone marrow cells from mice were cultured with recombinant mouse M-CSF (20 ng/ml) for generation of BMDMs. The primary MEFs were from day E13.5 old mouse embryos of *Irg1*^+/+^ or *Irg1*^-/-^ genotypes generated by cross-breeding *Irg1*^+/-^ heterozygous parents to provide MEFs. The BHK21, HEK293T, MEF, A549, 3T3 and MLE-12 cell lines were obtained from the American Type Culture Collection. All cells were cultured in DMEM or 1640 culture medium supplemented with 10% FBS (Gibco) in a 5% CO_2_ atmosphere at 37 °C. To block IFNAR1, we pretreated cells with 20 μg/ml of anti-IFNAR antibody clone MAR1-5A3 18 h before stimulating the cells, and we added fresh antibody upon stimulation.

### Viruses and virological assays

VSV was propagated and amplified by infection of 293 T cells. HSV-1 was propagated and amplified by infection of Vero cells. IAV was propagated by allantoic inoculation of embryonated hen’s eggs. Virus (VSV and HSV-1) titers of stocks and experimental samples were determined by TCID50 on BHK21 cells.

### Virus Infection

The cells were pretreated with OI (250 μM) or DMSO for 12 h, and infected with VSV (MOI = 1), VSV-GFP (MOI = 1), HSV-1 (MOI = 1) and IAV (MOI = 1) as described. Related genes and viral genomic RNA expression was analyzed at 8–12 h, and cytokines in the supernatant were analyzed at 24 h. For VSV in vivo infection, age- and sex-matched groups of littermate mice were intraperitoneally infected with VSV (1 × 10^7^ PFU/g) for 12 h. For IAV infection, mice were first anesthetized using tribromoethanol intraperitoneally (0.25 mg/g) and then intranasally inoculated with IAV (3 × 10^5^ PFU in 30 μl solution) for 5 days. For in vivo treatment, mice were i.p. pretreated with OI (50 mg/kg), DMM (50 mg/kg) or vehicle for 6 h.

To knockdown of GDI2 in the lungs of mice, the mice were anesthetized and administered adeno-associated virus (AAV) (50 μl; 1 × 10^13^ viral particles/ml; Vigene Biosciences, Shandong, China) containing GDI2 shRNA or scrambled shRNA via the intratracheal injection. After 7 days, the mice were sacrificed to evaluate the efficiency of GDI2 knockdown.

To generate bone marrow chimeric mice, CD45.1 mice were exposed to 8 Gy of X-ray radiation. After 24 h, 5 × 10^6^ *Irg1*^+/+^ or *Irg1*^−/−^ bone marrow cells were intravenously injected into irradiated mice. Six weeks after reconstitution, chimaera mice were infected with VSV and IAV.

For antibody-mediated neutrophil depletion, mice were administered an intraperitoneal injection of 170 mg of anti-Ly6G clone 1A8, anti-Gr-1 clone RB6-8C5, or isotype control clone 2A3 two days prior to infection and subsequently every three days. The confirmation of neutrophil depletion was achieved through flow cytometry, and judged by the CD45^+^CD11b^+^Ly6G^+^ cells.

### Quantitative Real-Time RT-PCR

Total RNA was extracted from cultured cells with TRIzol reagent according to the manufacturer’s instructions. RNA was reverse transcribed with Oligo (dT) primer for mRNA into cDNA with M-MLV Reverse Transcriptase (TaKaRa). RNA expression was quantified by real-time PCR with TB Green Premix Ex Taq (TaKaRa) and normalized to the level of β-actin according to the 2^−ΔΔCt^ calculation method. With the help of dissociation curve analysis and the sequencing of PCR products, pairs of specific primers of each cDNAs were designed and selected, without any primer dimers or unspecific amplification detected. Amplification of cDNA was performed on the ABI-Quant Studio 7 Flex. The sequences of the primers for quantitative real-time RT-PCR are listed in Supplementary Table. 1.

### RNA interference

PMs or MLE-12 cells were transfected with siRNA (20 nM) using siRNA transfection reagent. After 48 h, the cells were treated and infected as described. Cells were harvested and used for immunoblot or qRT–PCR. The mouse specific siRNA targeting *Irg1*, *Nrf2, Rab5, Rab7, Rab11, Gdi1, Gdi2* were designed and synthesized by GenePharma Co (Shanghai, China) and the siRNA targeting sequences are listed in Supplementary Table. 1.

### Immunoprecipitation and immunoblot analysis

Total proteins of cells were extracted with cell lysis buffer (CST) and additional protease inhibitor “cocktail” (Roche). The protein concentrations were quantified using a BCA assay, and equivalent amounts of proteins were utilized for immunoblot analysis. Cytoplasmic and plasma membrane fractions were isolated with Minute Plasma Membrane Protein Isolation and Cell Fractionation Kit according to the manufacturer’s instructions. Cytoplasmic and nuclear fractions were isolated with Nuclear and Cytoplasmic Protein Extraction Kit according to the manufacturer’s instructions.

For immunoprecipitation, HEK293T cells were grown to 70% confluence in 6-well plate, then transfected with GDI1-Myc, GDI2-Myc, Flag-Rab5 or Flag-Rab7 as described for 24 h. Subsequently, the cells were treated with OI (125 μM) or DMSO for 12 h. Cells were harvested and lysed with immunoprecipitation lysis buffer. Cell lysates were incubated with anti-Flag magnetic beads at 4 °C for 2 h. Magnetic beads were washed one time with NETN900 (20 mM Tris–HCl, pH 7.4; 0.1 mM EDTA; 0.5% NP-40; 900 mM NaCl) and two times with NETN100 (20 mM Tris–HCl, pH 7.4; 0.1 mM EDTA; 0.5% NP-40; 100 mM NaCl). Beads were boiled with immunoblot loading buffer for 10 min and subjected to immunoblot.

### Immunofluorescence and confocal microscopy

MLE-12 cells were seeded at a density of 2 × 10^5^ cells per ml in 6-well plates (2 ml per well) and subjected to the required treatments. Subsequently, cells were fixed in PBS solution containing 4% formaldehyde, permeabilized with 0.2% saporin along with a mixture of 5% BSA and 10% FCS. For immunofluorescence, cells were stained with indicated primary antibody and secondary antibody. For cholesterol staining, cells stained with filipin (50 μg/ml) for 2 h at room temperature. Images were obtained with laser scanning confocal microscope (Leica TCS SP8) and analyzed by the LAS X software version 2.0.2.15022. The JACoP plugin of ImageJ software was utilized for the quantification of colocalization.

### Validation of ITalk labeling

HEK293T cells were grown to 70% confluence in 6-well plate, then transfected with GDI1-Myc or GDI2-Myc plasmid using jetPEI transfection reagent for 24 h. Subsequently, the cells were treated with 100 μM ITalk or DMSO for 12 h. The cells were collected and centrifuged at 300 g for 5 min. The cell pellets were then resuspended in 400 μl ice-cold PBS containing EDTA-free Pierce Halt protease inhibitor cocktail. The cells were lysed by sonication on ice and the resulting lysates were collected by centrifugation at 12000 g for 15 min to remove debris. 50 μl lysate was separated out as input sample and the rest was incubated with 1 mM CuSO4, 100 mM TBTA, 100 mM Biotin-azide and 1 mM TCEP for 1 h at room temperature. The proteins were precipitated and washed to remove unbound biotin, then dissolved in 150 μl PBS containing 1.2% SDS via sonication and heating at 90 °C for 5 min. The samples were then diluted with 750 μl PBS for a final SDS concentration of 0.2%. The solutions were then incubated with 10 μl of streptavidin- magnetic beads for 2 h, followed by washing with 1 mL PBS three times. 40 μl loading buffer was added in the resulting beads and heated at 100 °C for10 min, centrifuged at 2000 g for 5 min and supernatant was collected as elution sample. The input and elution were resolved on SDS-PAGE gels, and the target protein was detected via Western Blot using monoclonal anti-Flag or anti-Myc antibody.

### Isolation of lung cell subpopulations

The lung tissue obtained from VSV-infected mice was sectioned into pieces and subsequently subjected to enzymatic digestion using a combination of collagenase-I, collagenase-IV, and DNase-I at 37 °C for 30 min. The obtained cells were stained with Anti-CD45 (percp-cy-5.5), Anti-Ly6G (FITC), Anti-CD64 (PE), and SiglecF (BV421). Subsequently, the specific cell subsets (at least 1 × 10^5^) were sorted using flow cytometry equipment (SH800, SONY). Total RNA was extracted from the sorted cell subsets using TRIzol reagent, followed by qRT-PCR analysis as described above. We defined CD45^+^Ly6G^+^CD64^-^ cells as neutrophils, CD45^+^CDLy6G^-^CD64^+^SiglecF^-^ cells as macrophages/monocytes and CD45^+^Ly6G^-^CD64^+^SiglecF^+^ cells as alveolar macrophages.

### Statistical analysis

Data are shown as mean ± SD and representative of three independent experiments (biological replicates) unless indicated otherwise in the figure legend. Statistical significance was assessed by unpaired two-tailed Student’s *t*-test with a value of *p* < 0.05 considered to be statistically significant (*p < 0.05, **p < 0.01, ***p < 0.001, N.S, not significant). Statistical analyses were carried out using GraphPad Prism 9 software. Image analysis was performed using ImageJ software. The illustrations were created using BioRender (https://app.biorender.com).

## Supplementary information

Supplementary Materials

unprocessed original images

## Data Availability

scRNA-seq data of VSV infected lung have been deposited in the NCBI GEO Database under accession code: GSE248329. RNA-seq data of *Irg1*^+/+^ and *Irg1*^-/-^ BMDMs have been deposited in the NCBI GEO Database under accession code: GSE249683. RNA-seq data of PMs treated with OI or DMSO has been deposited in the NCBI GEO Database under accession code: GSE249856.
