# Supplementary material for: Itaconate facilitates viral infection via alkylating GDI2 and retaining Rab GTPase on the membrane
Source: Signal Transduct Target Ther. 2024 Dec 27;9:371. doi: 10.1038/s41392-024-02077-8 (PMC11681089; doi:10.1038/s41392-024-02077-8)
Supplement: Supplementary file 1 — Supplementary Materials [file 41392_2024_2077_MOESM1_ESM.docx]

Supplementary Materials for

**Itaconate facilitates viral infection via alkylating GDI2 and retaining Rab GTPase on the membrane**

Shulei Yin ^1, 6^, Yijie Tao ^2, 6^, Tianliang Li ^1, 6^, Chunzhen Li ^1, 6^, Yani Cui ^2^, Yunyan Zhang ^3^, Shenhui Yin ^1^, Liyuan Zhao ^1^, Panpan Hu ^2^, Likun Cui ^1^, Yunyang Wu ^4^, Yixian He ^1^, Shu Yu ^1^, Jie Chen ^1^, Shaoteng Lu ^1^, Guifang Qiu ^1^, Mengqi Song ^1^, Qianshan Hou ^1^, Cheng Qian ^1^, Zui Zou ^1, 2, 5, *^, Sheng Xu ^1, *^, Yizhi Yu ^1, *^

Correspondence to:

Zui Zou (zouzui@smmu.edu.cn),

Sheng Xu (xusheng@immunol.org),

Yizhi Yu (yuyz@immunol.org)

**This PDF file includes:**

Supplementary Materials and Methods

Supplementary Figure. 1 to 10

Supplementary Table. 1

**Supplementary Materials and Methods**

**Metabonomics analysis**

The lungs of 8-week-old C57BL/6J mice were harvested after intraperitoneal infection with VSV for 12 hours or without infection. The lung metabonomics analysis of was performed using Q300 Kit (Metabo-Profile, Shanghai, China). Ultraperformance liquid chromatography coupled to tandem mass spectrometry (UPLC-MS/MS) system (ACQUITY UPLC-Xevo TQ-S, Waters Corp., Milford, MA, USA) was used to quantitate all targeted metabolites. For data processing, the raw data files generated by UPLC-MS/MS were processed using the iMAP platform (v1.0; Metabo-Profile, Shanghai, China).

**scRNA-sequencing sample preparation and acquisition**

8-weeks old C57BL/6J mice were i.p. infected with VSV. 12 hours after infection, mice were euthanized and their lungs were harvested. The tissue samples were first washed with phosphate-buffered saline (PBS), minced into small pieces on ice and enzymatically digested with Collagenase I (250 U/ml), Collagenase IV (100 U/ml), DNase I (30 U/ml) for 30 min at 37 ℃, with agitation. After digestion, samples were sieved through a 70 µm cell strainer, and centrifuged at 300 g for 5 min. After the supernatant was removed, the pelleted cells were suspended in red blood cell lysis buffer to lyse red blood cells. After washing with PBS containing 0.04 % BSA, the cell pellets were re-suspended in PBS containing 0.04 % BSA and re-filtered through a 35 μm cell strainer. Dissociated single cells were then stained with AO/PI for viability assessment.

BD Rhapsody system was used to capture the transcriptomic information of the single cells. Whole transcriptome libraries were prepared using the BD Rhapsody single-cell whole-transcriptome amplification workflow. Sequencing libraries were quantified using a High Sensitivity DNA chip (Agilent) on a Bioanalyzer 2200 and the Qubit High Sensitivity DNA assay (Thermo Fisher). All libraries were sequenced by DNBSEQ-T7 Sequencer (MGI, Shenzhen, China) on a 150 bp paired-end run. scRNA-seq data analysis was performed by NovelBio Co.,Ltd. with NovelBrain Cloud Analysis Platform.

**RNA sequencing analysis**

The BMDMs from *Irg1*^+/+^ and *Irg1*^-/-^ mice, or the PMs pretreated with OI and DMSO, were infected with VSV for 8 hours. Subsequently, the total RNA was isolated from the cells for further processing. cDNA library construction and sequencing were performed by Beijing Genomics Institute using the BGISEQ-500 platform. High-quality reads were aligned to the mouse reference genome GRCm38 (mm10). The expression levels for each of the genes were normalized to fragments per kilobase of exon model per million mapped reads (FPKM).

**FACS analysis of ROS**

Cells were plated at 1×10^6^ cells per ml in 12-well plates (1ml per well) and treated as required. 30 minutes before the end of the stimulation, the cell culture medium was changed with FCS-free DMEM containing CellROX detection reagent (1 μM). The cells were incubated at 37 ℃ for 30 minutes, subsequently washed with PBS, and gently scraped in PBS to prepare for flow cytometry analysis.

**Seahorse metabolic assays**

For real-time analysis of the ECAR and OCR, *Irg1*^+/+^ and *Irg1*^-/-^ macrophages were pretreated and infected as described, and then were analyzed with an XF-96 Extracellular Flux Analyzer (Seahorse Bioscience). The glycolytic stress test was performed using the following injection strategy, 25 mM glucose, 1 mM oligomycin, and 100 mM 2-DG. The mito-stress test was performed using serial injections of Oligomycin (2 μM), carbonyl cyanide-4- (trifluoromethoxy) phenylhydrazone (CCCP-2 μM), and Rotenone + Antimycin A (0.5 μM each).

**Cholesterol assay**

Cells were plated at 1×10^6^ cells per ml in 12-well plates (1ml per well) and treated as required. Total- and free-cholesterols were measured using Amplex Red Cholesterol Assay Kit according to the manufacturer’s instructions. Cholesterol concentrations were normalized by protein concentration detected by BCA assay in each individual sample.

**Click-IT geranylgeranylation assay**

MLE-12 cells were plated in 10 cm dishes. Cells were incubated with OI or DMSO and metabolic labeling with GGOH-azide (15 μM) for 24 hours in DMEM medium supplemented with 10 % FCS. Labeled cells were collected, frozen and the subsequent lysis of the frozen pellets was carried out with cell lysis buffer supplemented with protease inhibitor cocktail. The click reaction was performed using the Click labeling kit according to manufacturer’s instructions. Biotin-alkyne was used as substrate for this reaction. Proteins were precipitated and washed to remove unbound biotin, dissolved in 1× loading buffer and heated at 100 ℃ for 5 min. The sample were resolve on SDS-PAGE gels, and the geranylgeranyl-biotin complexes were detected via Western Blot using Streptavidin-HRP.

**Viral RNA purification and transfection**

VSV RNA was purified using the QIAamp viral RNA mini Kit according to the manufacturer’s instructions. Primary MEFs were plated at 2×10^5^ cells per ml in 24-well plates (0.5 ml per well) and treated as required. 250 ng of viral RNA per well was transfected using a transfection reagent Lipofectamine 2000 (Thermo Fisher). VSV RNA expression was detected by qRT-PCR. Results are then relative to those in the control group, set as 1.

**Analysis of GDI2 Modification by OI**

HEK293T cells were grown to 70 % confluence in 10 cm dishes, then transfected with GDI2-Flag plasmid using jetPEI transfection reagent. 24 hours after transfection, cells were treated with OI or DMSO for a further 12 hours. Following treatment, cells were washed with 5 mL PBS before lysis in 500 μ1 cell lysis buffer on ice. Plates were scraped and lysate transferred into microcentrifuge tubes. Flag-tagged GDI2 was immunoprecipitated using anti-Flag magnetic Beads. IP samples were subsequently centrifuged at 300 g for 2 minutes at 4 ℃, supernatant removed, and beads washed one time with NETN90 and two times with NETN100. The immune complexes were eluted by addition of 40 μl 1× loading buffer, boiled for 5 minutes and run on a 10 % gel. The resulting gel was stained using Coomassie Blue Staining Kit (Beyotime). The corresponding bands for GDI2-Flag were excised from the gel and subjected to in-gel digest.

For in-gel tryptic digestion, gel pieces were destained and dehydrated. Gel pieces were rehydrated with 10 ng/μl trypsin resuspended in 50 mM NH4HCO3 on ice for 1 h. Excess liquid was removed and gel pieces were digested with trypsin at 37 ℃ overnight. Peptides were extracted with 50 % acetonitrile/ 5 % formic acid, followed by 100 % acetonitrile. Peptides were dried to completion and resuspended in 2 % acetonitrile/ 0.1 % formic acid.

Samples were analyzed in an Orbitrap Exploris 480 coupled to an EASY-nLC 1200 UPLC system (Thermo Fisher). The resulting MS/MS data were processed using Proteome Discoverer 2.4. Tandem mass spectra were searched against target sequence (1 entry). Trypsin/P was specified as cleavage enzyme allowing up to 2 missing cleavages. Mass error was set to 10 ppm for precursor ions and 0.02 Da for fragment ions. Oxidation on Met, carbamidomethyl on Cys, acetylation on protein N-terminal and itaconation on Cys were specified as variable modification. Peptide confidence was set at high, and peptide ion score was set > 20.

**Supplementary Figures**


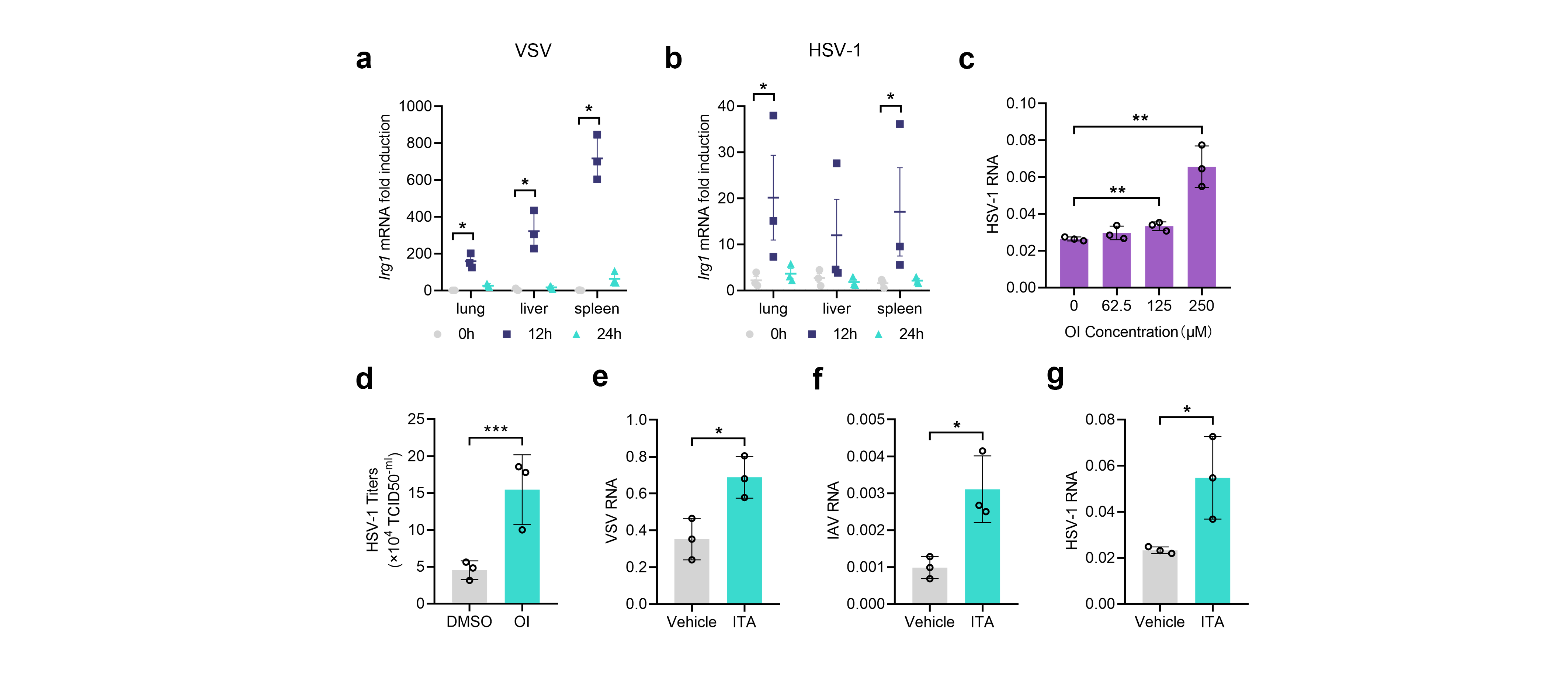


**Supplementary Fig. 1 Itaconate and OI facilitate viral infection. a, b** *Irg1* mRNA expression in the lungs, livers and spleens of mice i.p. infected with VSV (**a**) and HSV-1 (**b**) (n=3). **c** HSV-1 RNA in PMs pretreated with different concentrations of OI (n=3). **d** Viral titers in the supernatant of PMs pretreated with OI or DMSO (n=3). **e-g** Viral RNA in PMs pretreated with itaconate (ITA, 20mM) (n=3). Data are mean ± SD. **p* < 0.05, ***p* < 0.01, ****p* < 0.001 by an unpaired, two-tailed t-test.


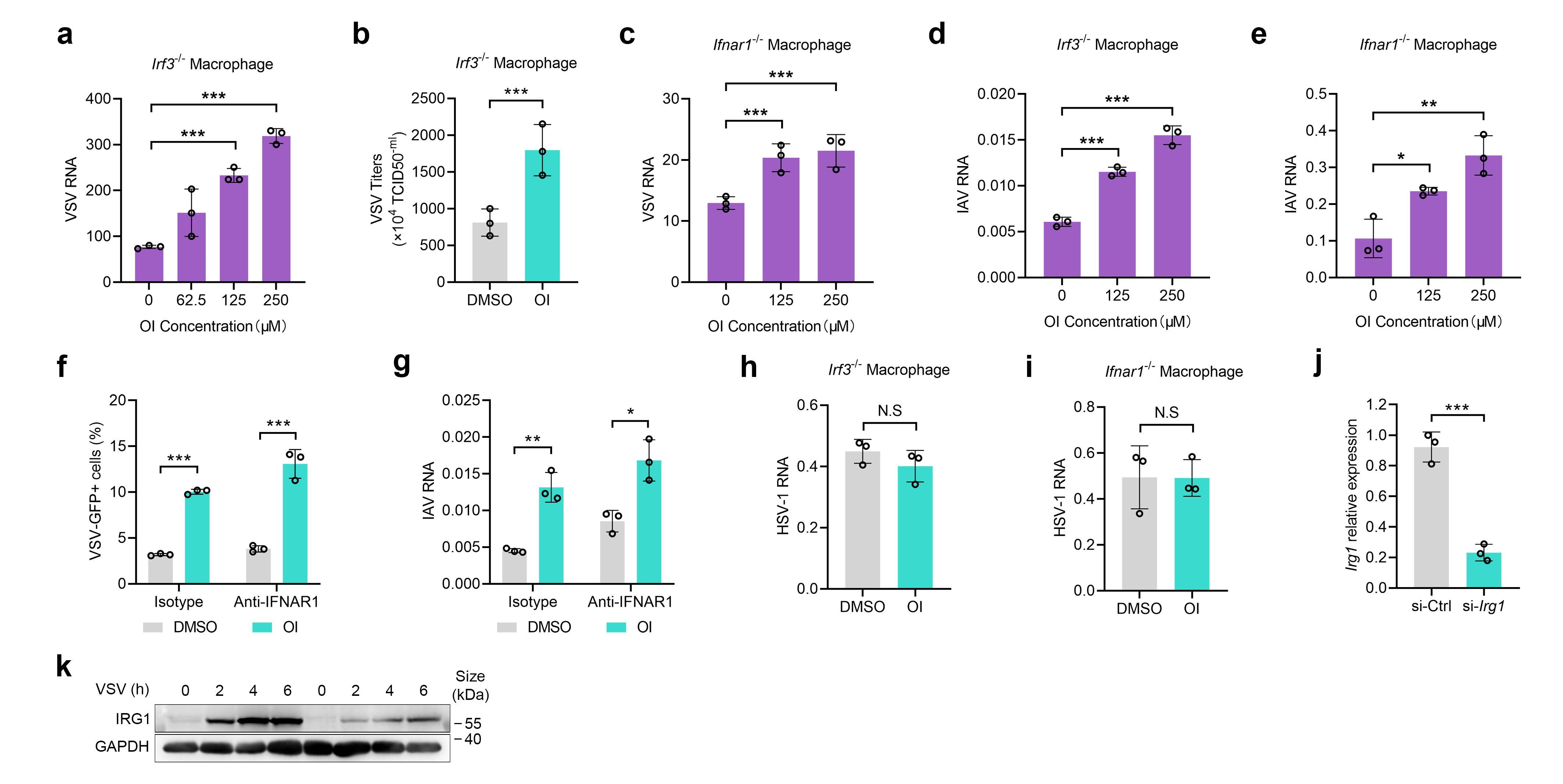


**Supplementary Fig. 2 OI promotes VSV and IAV infection independent of IFN-I pathway. a, b** VSV RNA (**a**) and titers (**b**) in *Irf3*-deficient PMs pretreated with OI (250 μM) or DMSO (n=3). **c** VSV RNA in *Ifnar1*-deficient PMs pretreated with OI or DMSO (n=3). **d, e** IAV RNA in *Irf3*- (**d**) and *Ifnar1*- (**e**) deficient PMs pretreated with OI or DMSO (n=3). **f, g** The effect of OI on VSV-GFP (**f**) and IAV (**g**) infection in MLE-12 cells pretreated with anti-mouse IFNAR-1 antibody (20 μg/ml) or isotype control antibody for 12 hours (n=3). **h, i** HSV-1 RNA in *Irf3*- (**h**) and *Ifnar1*- (**i**) deficient PMs pretreated with OI or DMSO (n=3). **j** Relative RNA expression of *Irg1* in *Irf3*^-/-^ PMs carrying *Irg1* or control siRNA. **k** Immunoblot of IRG1 in *Irf3*^-/-^ PMs carrying *Irg1* or control siRNA infected with VSV. Data are mean ± SD. **p* < 0.05, ***p* < 0.01, ****p* < 0.001, N.S, not significant by an unpaired, two-tailed t-test.


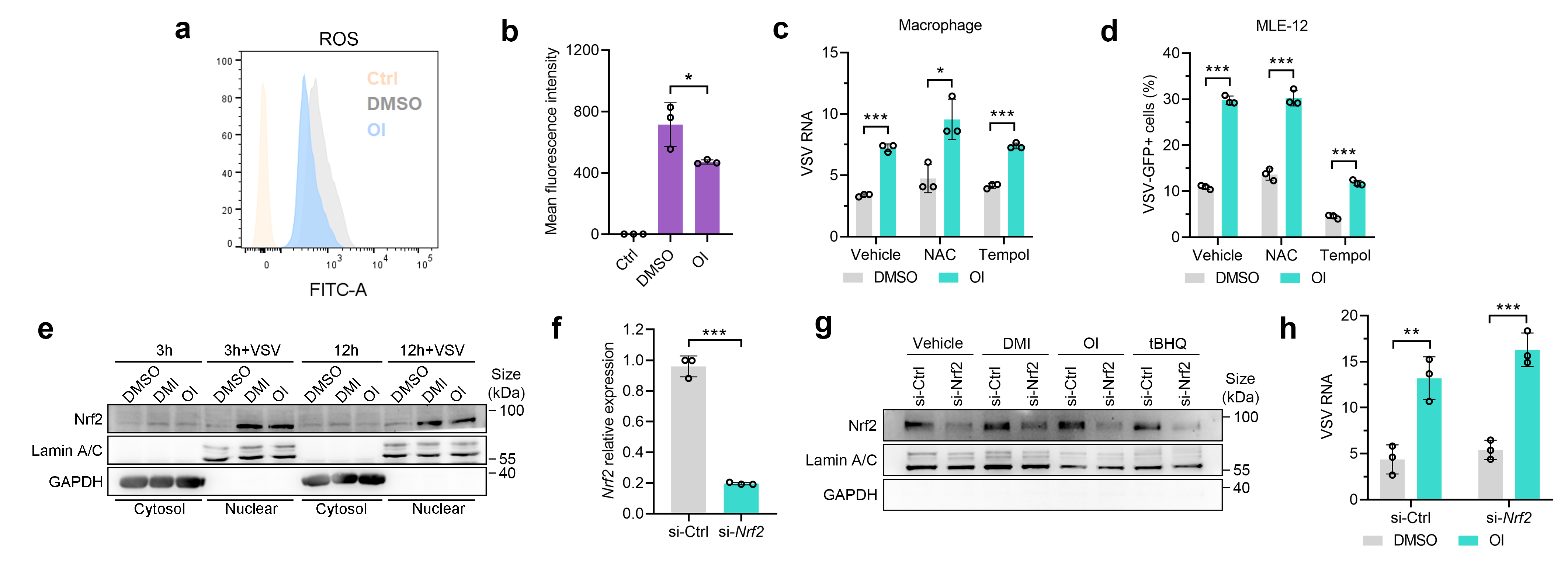


**Supplementary Fig. 3 Antioxidant reaction is not responsible for OI induced viral overgrowth. a, b** ROS production in PMs pretreated with OI (250 μM) or DMSO, infected with VSV (n=3). **c** The effect of OI on VSV infection in PMs pretreated with NAC (2 mM), Tempol (2 mM) or vehicle (n=3). **d** The effect of OI on VSV-GFP infection rate in MLE-12 cells pretreated as in (**c**) (n=3). **e** Immunoblot of Nrf2 in the nuclear and cytosol fractions of PMs pretreated with DMI (250 μM), OI (250 μM) or DMSO, infected with VSV. **f** Relative RNA expression of *Nrf2* in PMs carrying *Nrf2* or control siRNA. **g** Immunoblot of Nrf2 in the nuclear fractions of PMs carrying *Nrf2* or control siRNA, treated with DMI, OI, tBHQ (10 μM) or DMSO. **h** VSV RNA in PMs carrying *Nrf2* or control siRNA, pretreated with OI or DMSO (n=3). Data are mean ± SD or representative of 3 independent experiments with similar results. **p* < 0.05, ***p* < 0.01, ****p* < 0.001 by an unpaired, two-tailed t-test.

**
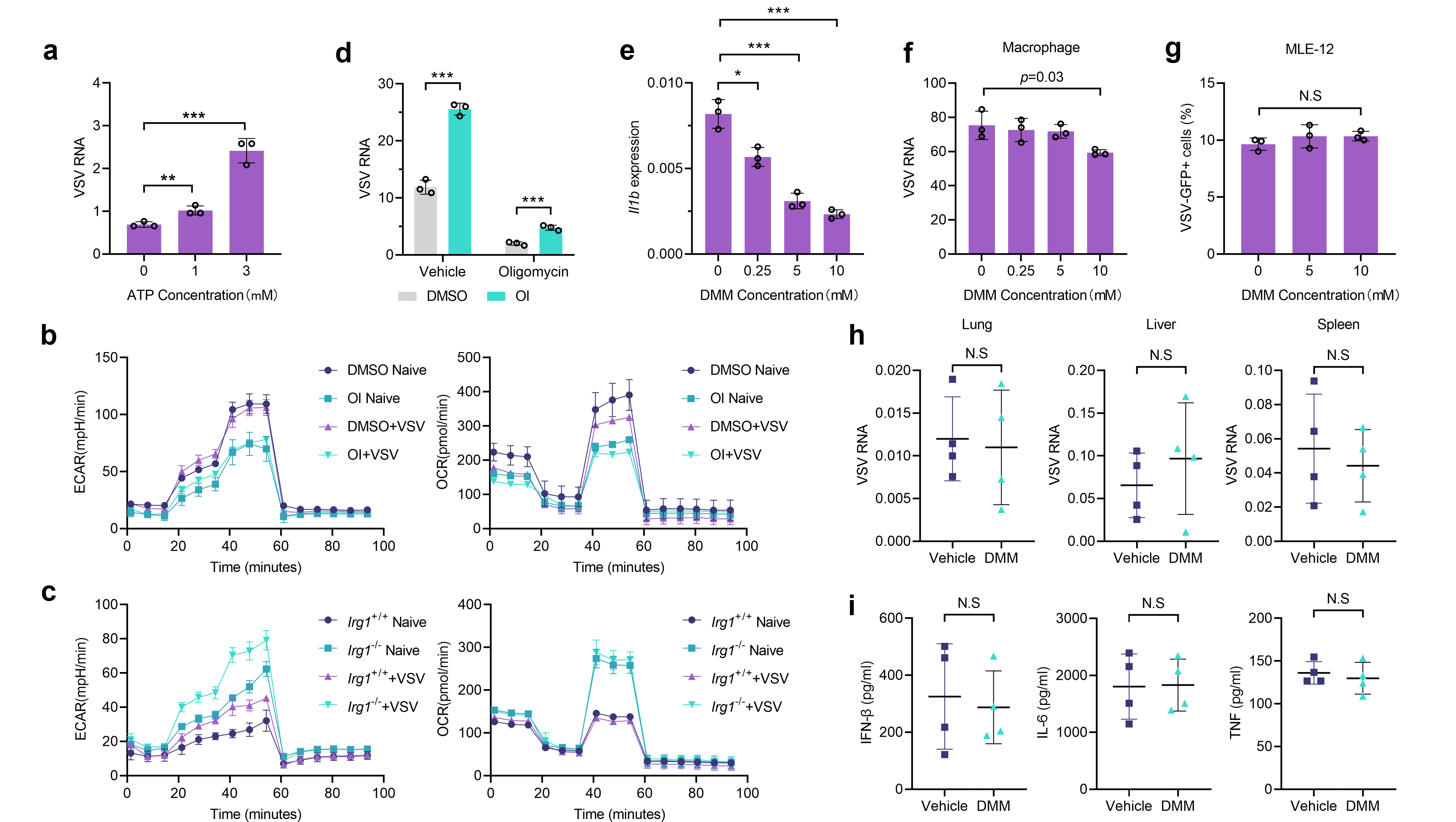
**

**Supplementary Fig. 4 OI does not regulate VSV infection via modulation of glucose metabolism. a** VSV RNA in PMs pretreated with different concentrations of ATP (0, 1 or 3 mM) (n=3). **b** Seahorse analysis of ECAR and OCR in PMs pretreated with OI (250 μM) or DMSO, infected with VSV or not (n=3). **c** Seahorse analysis of ECAR and OCR in *Irg1*^+/+^ and *Irg1*^-/-^ BMDMs infected with VSV or not (n=3). **d** The effect of OI on VSV RNA in PMs pretreated with oligomycin (5 μM) or vehicle (n=3). **e** *Il1b* mRNA expression in PMs pretreated different concentrations of DMM (0, 0.25, 5 or 10 mM), infected with VSV to confirm the efficacy of DMM (n=3). **f** VSV RNA in PMs pretreated as in (**e**) (n=3). **g** VSV-GFP infection rate in MLE-12 cells pretreated with DMM (0, 5 or 10 mM) (n=3). **h** VSV RNA in the lungs, spleens and livers of mice i.p. pretreated with DMM (50 mg/kg) or vehicle (n=4). **i** ELISA analysis of IFN-β, IL-6 and TNF-α in the serum of mice in (**h**) (n=4). Data are mean ± SD. **p* < 0.05, ***p* < 0.01, ****p* < 0.001, N.S, not significant by an unpaired, two-tailed t-test.


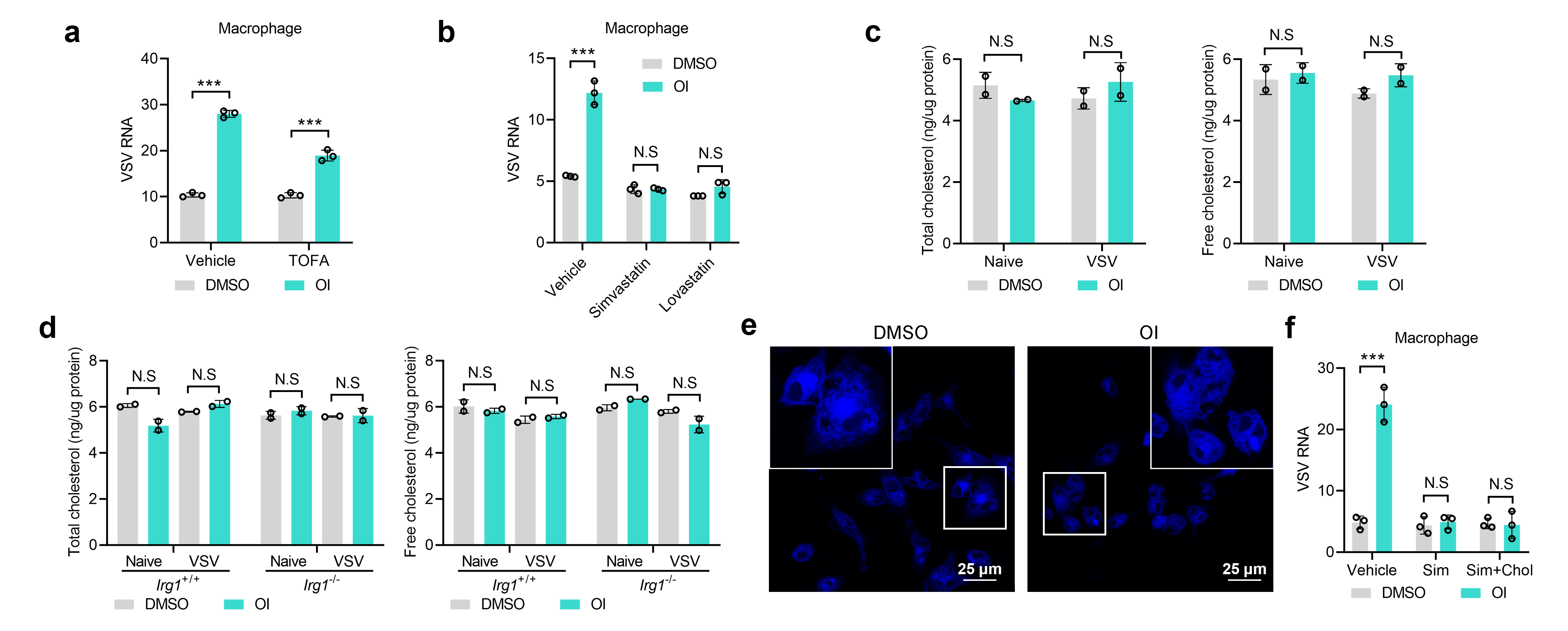


**Supplementary Fig. 5 OI induced VSV overgrowth is independent of cholesterol. a** The effect of OI on VSV RNA in PMs pretreated with TOFA (30 μM)) or vehicle (n=3). **b** The effect of OI on VSV RNA in PMs pretreated with simvastatin (10 μM), lovastatin (20 μM) or vehicle (n=3). **c** Total- and free- cholesterol concentrations in MLE-12 cells pretreated with OI or DMSO, infected with VSV or not (n=2). **d** Total- and free- cholesterol concentrations in *Irg1*^+/+^ and *Irg1*^-/-^ BMDMs pretreated with OI or DMSO, infected with VSV or not (n=2). **e** Confocal analysis of intracellular cholesterol distribution in PMs pretreated with OI or DMSO. **f** The effect of OI on VSV RNA in PMs pretreated with simvastatin (Sim, 10 μM) with or without a cell permeable cholesterol (Chol, 5 ug/ml) (n=3). Data are mean ± SD or representative of 3 independent experiments with similar results. ****p* < 0.001, N.S, not significant by an unpaired, two-tailed t-test.


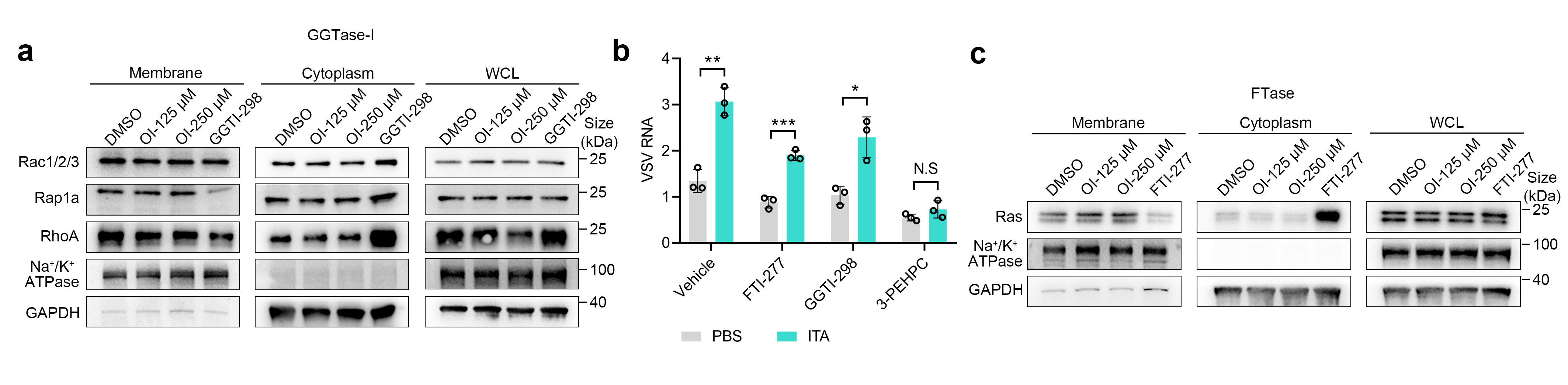


**Supplementary Fig. 6 The influence of OI on the subcellular localization of GGTase-I and FTase target proteins. a** Immunoblot of GGTase-I target proteins in the membrane, cytoplasm and total cell fractions of MLE-12 cells pretreated with OI, GGTI-298 (10 μM) or DMSO. **b** VSV infection in PMs pretreated with ITA (10 mM) or PBS plus FTI-277 (10 μM), GGTI-298 (10 μM), 3-PEHPC (1.5 mM) or vehicle for 12 hours (n=3). **c** Immunoblot of FTase target protein in the membrane, cytoplasm and total cell fractions of MLE-12 cells pretreated with OI, FTI-277 (10 μM) or DMSO. Data are mean ± SD or representative of 3 independent experiments with similar results. **p* < 0.05, ***p* < 0.01, ****p* < 0.001, N.S, not significant by an unpaired, two-tailed t-test.


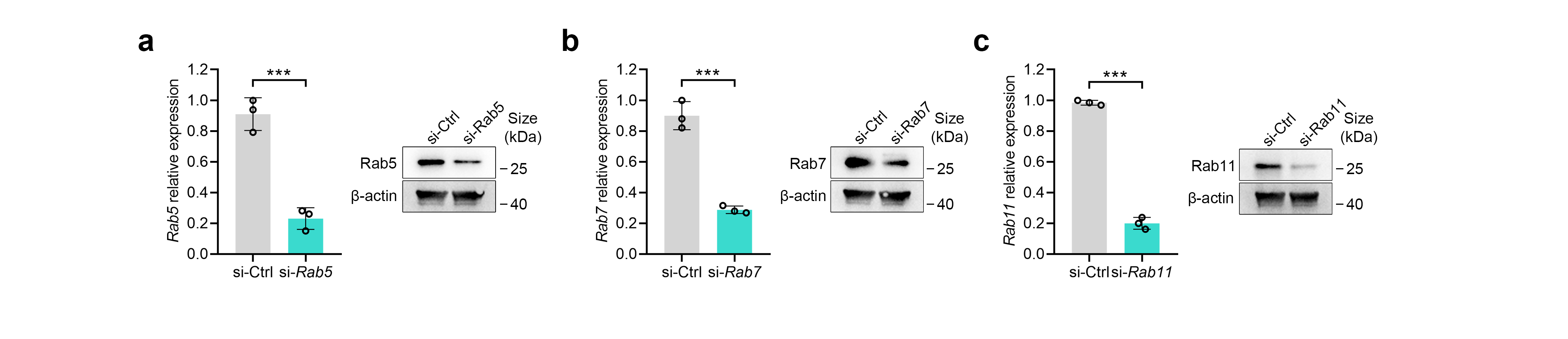


**Supplementary Fig. 7 The knockdown efficiency for Rab proteins. a-c** RNA and protein expression of Rabs in MLE-12 cells carrying Rab5, Rab7, Rab11 or control siRNA. Data are mean ± SD or representative of 3 independent experiments with similar results. ****p* < 0.001 by an unpaired, two-tailed t-test.


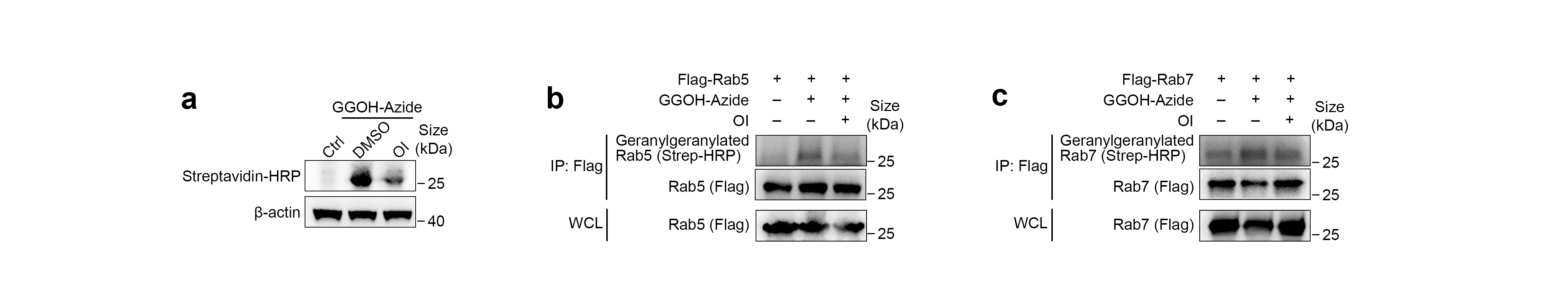


**Supplementary Fig. 8 OI impairs protein geranylgeranylation on Rabs. a** Immunoblot of geranylgeranylated proteins in MLE-12 cells treated with GGOH-azide (15 μM), OI (125 μM) or DMSO as indicated. **b, c** Immunoblot of geranylgeranylated Rab5 (**b**) and Rab7 (**c**) in HEK293T cells transfected with Flag-Rab5 (**b**) or Flag-Rab7 (**c**), treated with GGOH-azide, OI or DMSO as indicated. Data are representative of 3 independent experiments with similar results.


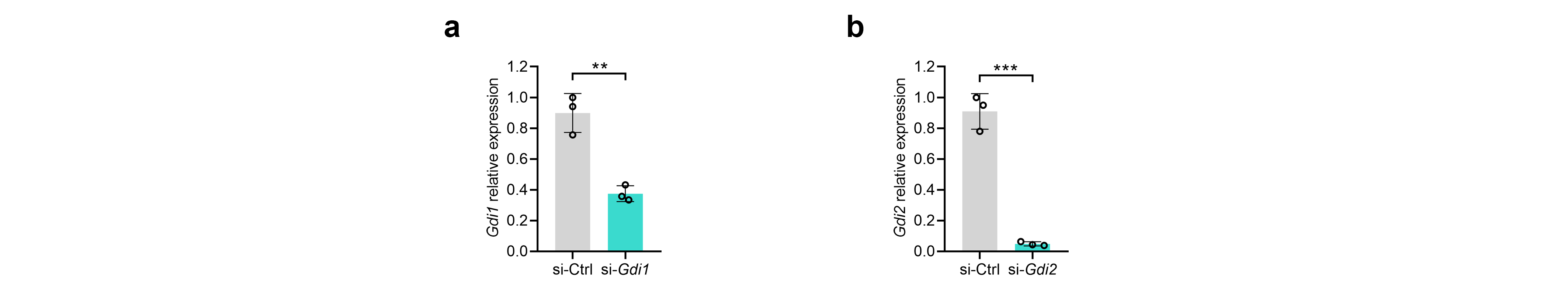


**Supplementary Fig. 9 The knockdown efficiency for GDIs.** **a, b** Relative RNA expression of GDIs in MLE-12 cells carrying *Gdi1*, *Gdi2* or control siRNA. Data are mean ± SD. ***p* < 0.01, ****p* < 0.001 by an unpaired, two-tailed t-test.


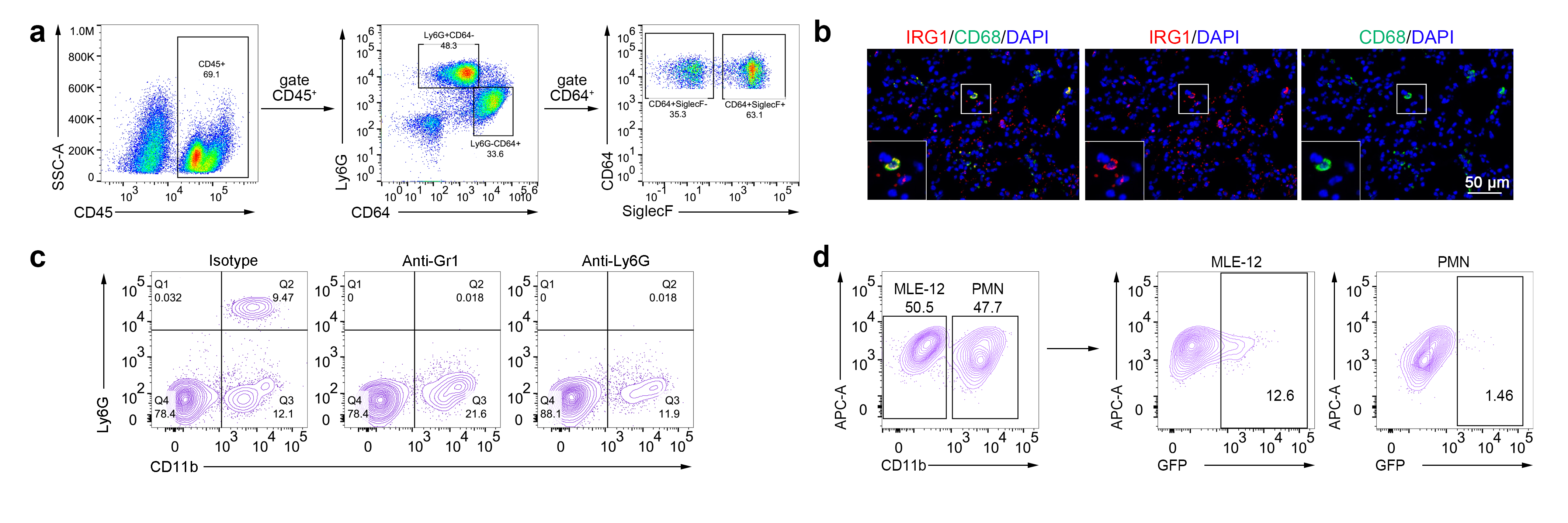


**Supplementary Fig. 10 *Irg1* is predominantly expressed by neutrophils in VSV infected lung. a** The sorting procedure for obtaining the cell populations in **Fig. 6d**. **b** Immunofluorescence analysis of IRG1 and CD68 expression in VSV infected lung. **c** Neutrophils (CD45^+^CD11b^+^Ly6G^+^) percentage in the peripheral blood of mice i.p. treated with anti-Ly6G, anti-Gr1 or isotype control antibody for 2 days. **d** VSV-GFP infection rate in MLE-12 cells and neutrophils (PMN) distinguished by CD11b labeling. Data are representative of 3 independent experiments with similar results.

**Supplementary Table. 1**

Key resources table

| **REAGENT or RESOURCE** | **SOURCE** | **IDENTIFIER** |
| --- | --- | --- |
| **Antibodies** | | |
| Anti-RIG-I | Cell Signaling Technology | Cat# 3743; RRID:AB_2269233 |
| Anti-MAVS | Cell Signaling Technology | Cat# 4983; RRID:AB_823566 |
| Anti-TBK1 | Cell Signaling Technology | Cat# 3504; RRID:AB_2255663 |
| Anti-p-TBK1 | Cell Signaling Technology | Cat# 5483; RRID:AB_10693472 |
| Anti-IRF-3 | Cell Signaling Technology | Cat# 4302; RRID:AB_1904036 |
| Anti-p-IRF-3 | Cell Signaling Technology | Cat# 4947; RRID:AB_823547 |
| Anti-STAT1 | Cell Signaling Technology | Cat# 14994; RRID:AB_2737027 |
| Anti-p-STAT1 | Cell Signaling Technology | Cat# 9167; RRID:AB_561284 |
| Anti-GAPDH | Cell Signaling Technology | Cat# 2118; RRID:AB_561053 |
| Anti-NRF2 | Cell Signaling Technology | Cat# 12721; RRID:AB_2715528 |
| Anti-Lamin A/C | Cell Signaling Technology | Cat# 2032; RRID:AB_2136278 |
| Anti-Na, K ATPase | Cell Signaling Technology | Cat# 3010; RRID:AB_2060983 |
| Anti-β-actin | Cell Signaling Technology | Cat# 4970; RRID:AB_2223172 |
| Anti-IRG1 | Cell Signaling Technology | Cat# 19857; RRID: AB_3076520 |
| Anti-CD68 | Cell Signaling Technology | Cat# 97778; RRID:AB_2928056 |
| Anti-Rac1/2/3 | Cell Signaling Technology | Cat# 2465; RRID:AB_2176152 |
| Anti-Rap1a | Cell Signaling Technology | Cat# 2399; RRID:AB_2284915 |
| Anti-RhoA | Cell Signaling Technology | Cat# 2117; RRID:AB_10693922 |
| Anti-Rab5 | Cell Signaling Technology | Cat# 3547; RRID:AB_2300649 |
| Anti-Rab7 | Cell Signaling Technology | Cat# 95746; RRID:AB_2800252 |
| Anti-RAS | Cell Signaling Technology | Cat# 67648; RRID:AB_2910195 |
| Anti-EEA1 | Cell Signaling Technology | Cat# 48453; RRID:AB_2920538 |
| Anti-Myc-Tag | Cell Signaling Technology | Cat# 2276; RRID:AB_331783 |
| Anti-Flag-Tag | Cell Signaling Technology | Cat# 14793; RRID:AB_2572291 |
| Anti-Rab1a | Proteintech | Cat# 11671-1-AP; RRID:AB_2173437 |
| Anti-Rab6 | Proteintech | Cat# 10187-2-AP; RRID:AB_2175463 |
| Anti-Rab11 | Proteintech | Cat# 15903-1-AP; RRID:AB_2173458 |
| Anti-GDI2 | Proteintech | Cat# 10116-1-AP; RRID:AB_2279073 |
| Anti-Rab5 | Abcam | Cat# ab218624; RRID:AB_2892717 |
| Anti-LAMP1 | Abcam | Cat# ab208943; RRID:AB_2923327 |
| Anti-RabGGTB | Abcam | Cat# ab187717; RRID:AB_2751007 |
| Anti-mouse IFNAR1 | BioXcell | Cat# BE0241; RRID:AB_2687723 |
| Anti-mouse Ly6G | BioXcell | Cat# BE0075-1; RRID:AB_1107721 |
| Anti-mouse Gr-1 | BioXcell | Cat# BE0075; RRID:AB_10312146 |
| **Bacterial and virus strains** | | |
| Vesicular stomatitis virus (VSV) | ATCC | Cat# VR-1238 |
| Vesicular stomatitis virus (VSV)-GFP | This paper | N/A |
| Herpes simplex virus (HSV-1) | Provided by Dr. Qihan Li | N/A |
| Influenza A virus PR8 strain (IAV) | ATCC | Cat# VR-1469 |
| **Chemicals, peptides, and recombinant proteins** | | |
| Trizol | Thermo Fisher | Cat# 15596018 |
| ATP solution | Thermo Fisher | Cat# R0441 |
| Dynabeads M-270 Streptavidin | Thermo Fisher | Cat# 65305 |
| GGOH-azide | Thermo Fisher | Cat# C10249 |
| Biotin Alkyne | Thermo Fisher | Cat# B10185 |
| Itaconic acid | Sigma Aldrich | Cat# I29204 |
| Dimethyl itaconate (DMI) | Sigma Aldrich | Cat# 109533 |
| poly I:C | Sigma Aldrich | Cat# P1530 |
| Dimethyl itaconate (DMM) | Sigma Aldrich | Cat# 109533 |
| TOFA | Sigma Aldrich | Cat# T6575 |
| Geranylgeraniol (GGOH) | Sigma Aldrich | Cat# G3278 |
| Farnesol (FOH) | Sigma Aldrich | Cat# F203 |
| Squalene (SQE) | Sigma Aldrich | Cat# S3626 |
| N-Acetyl-L-cysteine (NAC) | Sigma Aldrich | Cat# 9165 |
| tBHQ | Sigma Aldrich | Cat# 07293 |
| Filipin | Sigma Aldrich | Cat# SAE0087 |
| Cholesterol-Water Soluble | Sigma Aldrich | Cat# C4951 |
| Anti-FLAG Magnetic Beads | Sigma Aldrich | Cat# M8823 |
| 4-Octyl Itaconate (OI) | MCE | Cat# HY-112675 |
| ITalk | MCE | Cat# HY-133870 |
| 3-PEHPC | MCE | Cat# HY-16011 |
| Biotin-azide | MCE | Cat# HY-129832 |
| TCEP | MCE | Cat# HY-W011500 |
| TBTA | MCE | Cat# HY-116677 |
| Simvastatin | Selleck | Cat# S1792 |
| Lovastatin | Selleck | Cat# S2061 |
| GGTI-298 | Selleck | Cat# S7466 |
| FTI-277 | Selleck | Cat# S7465 |
| Tempol | Selleck | Cat# S2910 |
| Oligomycin | Selleck | Cat# S1478 |
| Recombinant Murine M-CSF | PeproTech | Cat# 315-02 |
| Dimethyl malonate | Sinopharm Chemical Reagent | Cat# 30069626 |
| CuSO4 | Sangon Biotech | Cat# A603008 |
| HRP-conjugated Streptavidin | Proteintech | Cat# SA00001-0 |
| DNA Transfection Reagent | Ployplus | Cat# 101000020 |
| siRNA transfection reagent | Ployplus | Cat# 101000028 |
| Viral RNA transfection reagent | Thermo Fisher | Cat# 11668030 |
| **Critical commercial assays** | | |
| Nuclear and Cytoplasmic Protein Extraction Kit | Beyotime Biotech | Cat# P0027 |
| Mouse IFN-β ELISA Kit | Biolegend | Cat# 439408 |
| Cholesterol Assay Kit | Thermo Fisher | Cat# A12216 |
| Click-it Protein Reaction Buffer Kit | Thermo Fisher | Cat# C10276 |
| Fluorometric Intracellular ROS Kit | Thermo Fisher | Cat# C10492 |
| Mouse IL-6 ELISA Kit | R&D systems | Cat# SM6000B |
| Mouse TNF-alpha ELISA Kit | R&D systems | Cat# SMTA00B |
| Neutrophil Selection Kit | Stemcell | Cat# 19762 |
| Plasma Membrane Protein Isolation and Cell Fractionation Kit | Invent | Cat# SM-005 |
| QIAamp Viral RNA Mini Kit | QIAGEN | Cat# 52906 |
| **Deposited data** | | |
| scRNA-seq of VSV infected lung | This paper | GSE248329 |
| RNA-seq of *Irg1*^+/+^ and *Irg1*^-/-^ BMDMs | This paper | GSE249683 |
| RNA-seq of PMs treated with OI or DMSO | This paper | GSE249856 |
| Unprocessed scans of all blots and all values used to generate graphs | This paper | Data S1. |
| **Experimental models: Cell Lines** | | |
| BHK21 | ATCC | Cat # CCL-10; RRID:CVCL_1915 |
| MEF | ATCC | Cat# SCRC-1008; RRID:CVCL_9115 |
| 3T3 | ATCC | Cat# CRL-1658; RRID:CVCL_0594 |
| A549 | ATCC | Cat# CCL-185; RRID:CVCL_0023 |
| MLE-12 | ATCC | Cat# CRL-2110; RRID:CVCL_3751 |
| **Experimental models: Organisms/Strains** | | |
| C57BL/6 mice | Joint Ventures Sipper BK Experimental Animals | N/A |
| *Irg1*^-/-^ C57BL/6J mice | Provided by Dr. Yingke Li | N/A |
| *Irf3*^-/-^ C57BL/6J mice | Provided by Dr. Tadatsugu Taniguchi | N/A |
| *Ifnar1*^-/-^ C57BL/6 mice | The Jackson Laboratory | RRID:IMSR_JAX:028288 |
| **Oligonucleotides** | | |
| siRNA targeted sequence: Irg1 (5’-3’):  CACCUUGUGACACCAGAAA | Genepharma | N/A |
| siRNA: Nrf2 sense (5’-3’):  CCGAAUUACAGUGUCUUAA | Genepharma | N/A |
| siRNA targeted sequence: Rab5 (5’-3’):  GCAGAUGACAACAGCUUAU | Genepharma | N/A |
| siRNA targeted sequence: Rab7 (5’-3’):  CUCUCAUGAACCAGUAUGU | Genepharma | N/A |
| siRNA targeted sequence: Rab11 (5’-3’):  GUGCCUUAUUGGUUUAUGA | Genepharma | N/A |
| siRNA targeted sequence: Gdi1 (5’-3’):  GUGGCUAUCAGUGAUUUAU | Genepharma | N/A |
| siRNA targeted sequence: Gdi2 (5’-3’):  GCUCUUGGAACCAAUUGAA | Genepharma | N/A |
| Primer: β-actin (FWD):  AGTGTGACGTTGACATCCGT | This paper | N/A |
| Primer: β-actin (REV):  GCAGCTCAGTAACAGTCCGC | This paper | N/A |
| Primer: Irg1 (FWD):  AATGAAACCTTGGGTCTTATGCC | This paper | N/A |
| Primer: Irg1 (REV):  TGCCCATGACTTATCCAGACAG | This paper | N/A |
| Primer: Ifnb1 (FWD):  CCCTATGGAGATGACGGAGA | This paper | N/A |
| Primer: Ifnb1 (REV):  TCCCACGTCAATCTTTCCTC | This paper | N/A |
| Primer: Tnf (FWD):  GCCTCTTCTCATTCCTGCTT | This paper | N/A |
| Primer: Tnf (REV):  TGGGAACTTCTCATCCCTTTG | This paper | N/A |
| Primer: Il6 (FWD):  CCGGAGAGGAGACTTCACAG | This paper | N/A |
| Primer: Il6 (REV):  TCCACGATTTCCCAGAGAAC | This paper | N/A |
| Primer: Isg15 (FWD):  GGTGTCCGTGACTAACTCCAT | This paper | N/A |
| Primer: Isg15 (REV):  TGGAAAGGGTAAGACCGTCCT | This paper | N/A |
| Primer: Mx1 (FWD):  GACCATAGGGGTCTTGACCAA | This paper | N/A |
| Primer: Mx1 (REV):  AGACTTGCTCTTTCTGAAAAGCC | This paper | N/A |
| Primer: HSV TK (FWD):  AGTTGCGTGGTGGTGGTTT | This paper | N/A |
| Primer: HSV TK (REV):  ACAAAAAGCCACGGAAGTCC | This paper | N/A |
| Primer: VSV (FWD):  ACGGCGTACTTCCAGATGG | This paper | N/A |
| Primer: VSV (REV):  CTCGGTTCAAGATCCAGGT | This paper | N/A |
| Primer: IAV (FWD):  TCAGGCCCCCTCAAAGCCGA | This paper | N/A |
| Primer: IAV (REV):  GGGCACGGTGAGCGTGAACA | This paper | N/A |
| Primer: Nrf2 (FWD):  ATCAGGCCCAGTCCCTCAAT | This paper | N/A |
| Primer: Nrf2 (REV):  CCAGCGAGGAGATCGATGAG | This paper | N/A |
| **Software and algorithms** | | |
| Leica laser scanning software | Leica | http://www.leica-microsystems.com/products/confocal-microscopes/details/product/leica-tcs-sp8/ |
| Flowjo_v10.8 | Flowjo | https://www.flowjo.com/ |
| Image J | Image J | https://imagej.nih.gov/ij/download.html |
| Prism 9 | Graphpad software | https://www.graphpad.com |
