# Supplementary material for: Itaconate facilitates viral infection via alkylating GDI2 and retaining Rab GTPase on the membrane
Source: Signal Transduct Target Ther. 2024 Dec 27;9:371. doi: 10.1038/s41392-024-02077-8 (PMC11681089; doi:10.1038/s41392-024-02077-8)

unprocessed original images

Fig. 2c

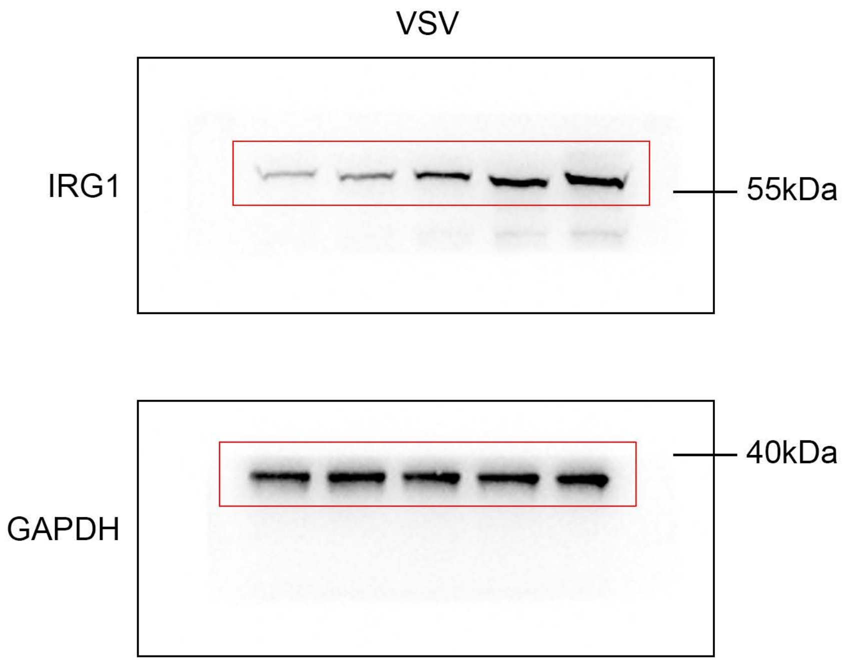

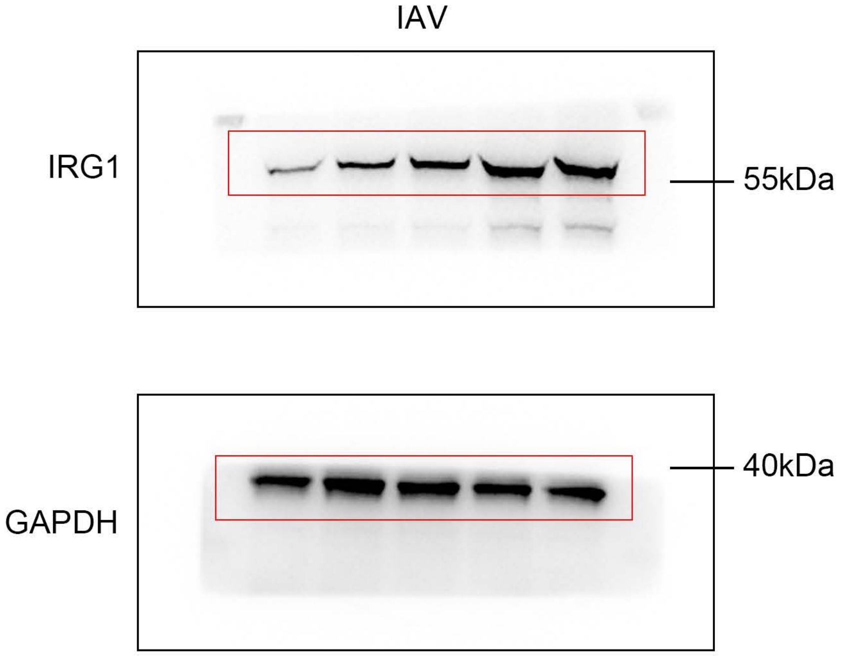

Fig. 2i

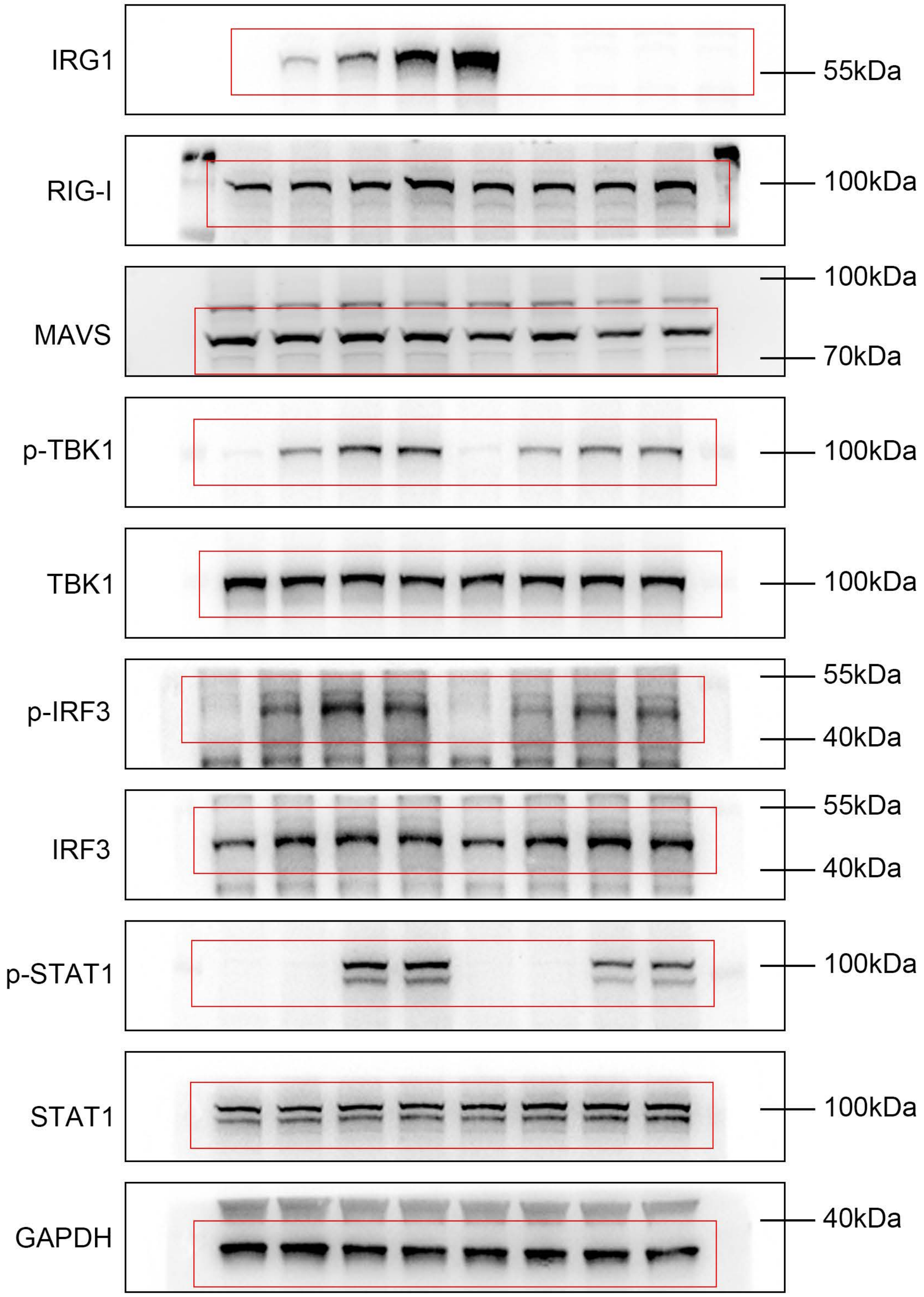
IRG1
55kDa
RIG-I
100kDa
MAVS
100kDa
70kDa
p-TBK1
100kDa
TBK1
100kDa
p-IRF3
55kDa
40kDa
IRF3
55kDa
40kDa
p-STAT1
100kDa
STAT1
100kDa
GAPDH
40kDa

Fig. 4a

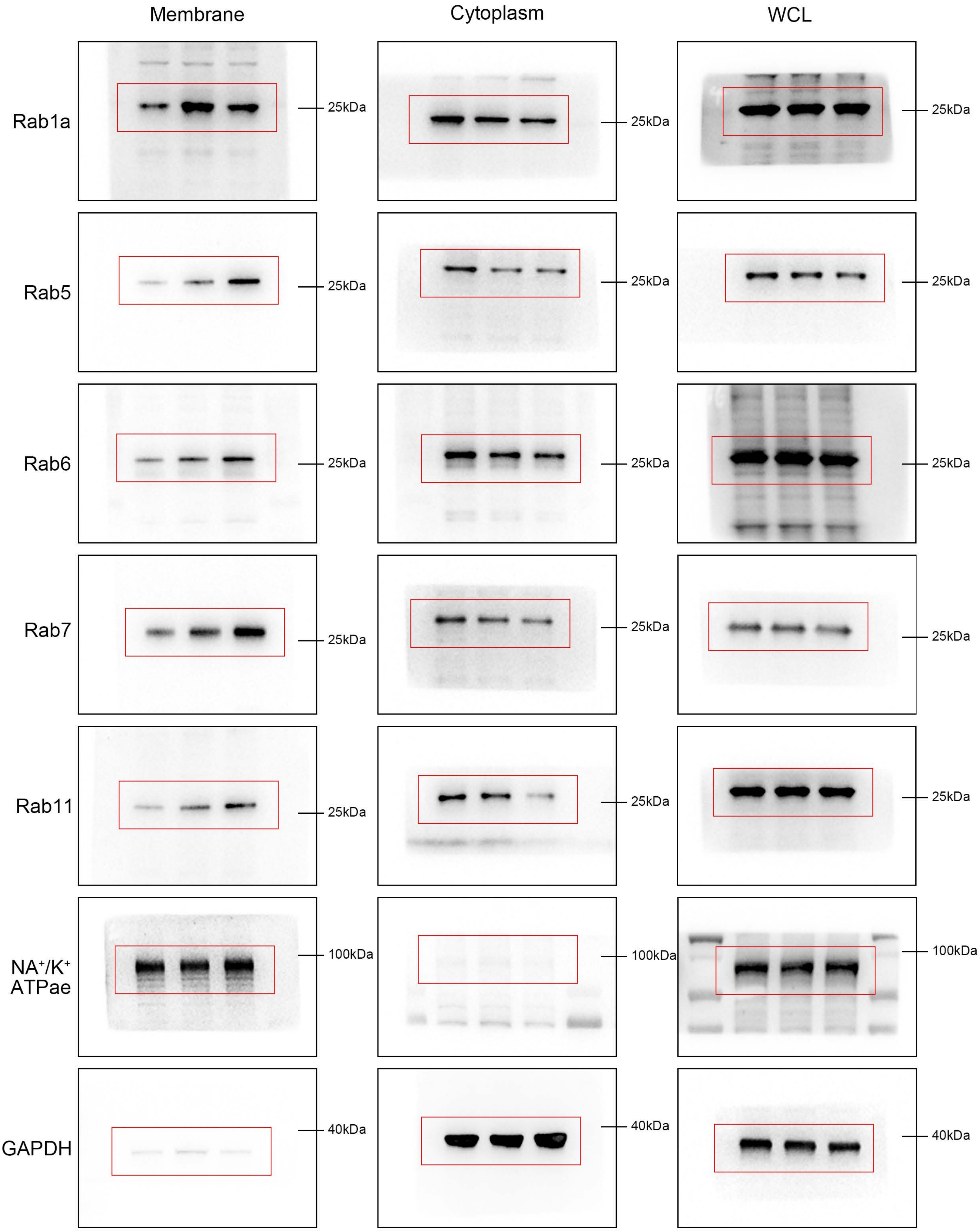

Membrane
Cytoplasm
WCL
Rab1a
Rab5
Rab6
Rab7
Rab11
NA+/K+ ATPae
GAPDH
25kDa
100kDa
40kDa

Fig. 4c

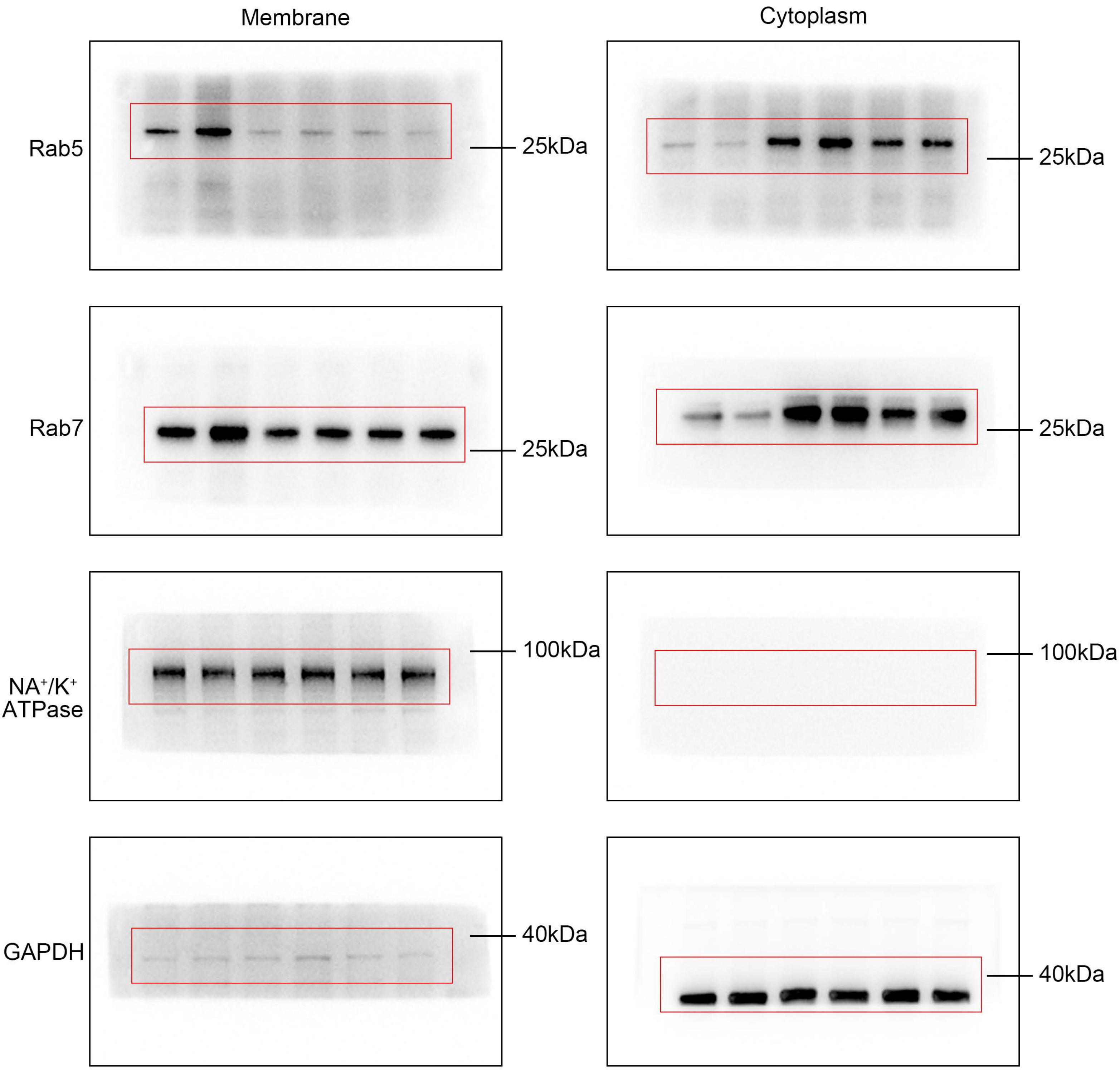
Membrane
Cytoplasm
Rab5
25kDa
25kDa
Rab7
25kDa
25kDa
NA+/K+ ATPase
100kDa
100kDa
GAPDH
40kDa
40kDa

Fig. 5d

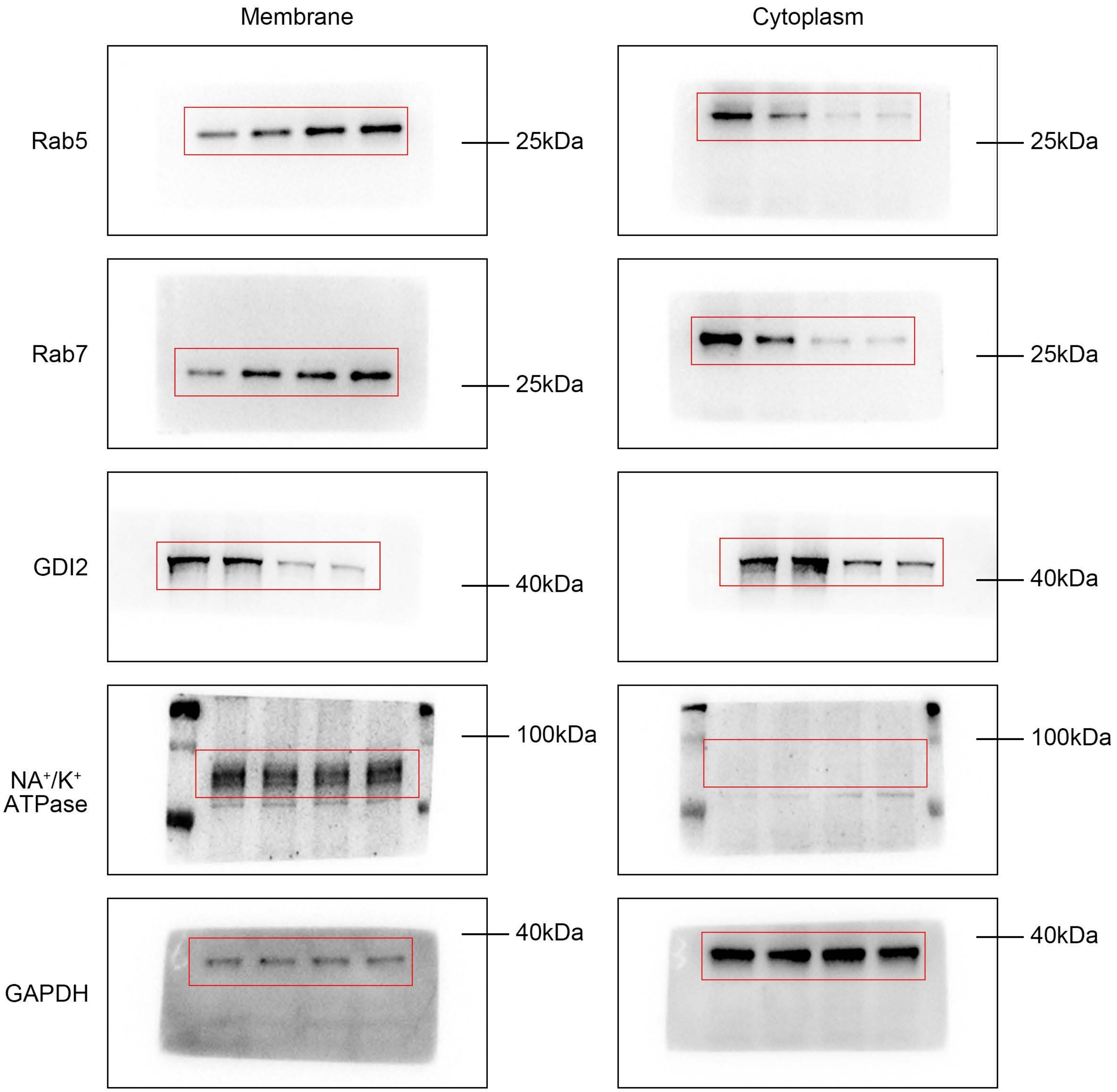

Membrane
Cytoplasm
Rab5
25kDa
25kDa
Rab7
25kDa
25kDa
GDI2
40kDa
40kDa
NA⁺/K⁺ ATPase
100kDa
100kDa
GAPDH
40kDa
40kDa

Fig. 5e

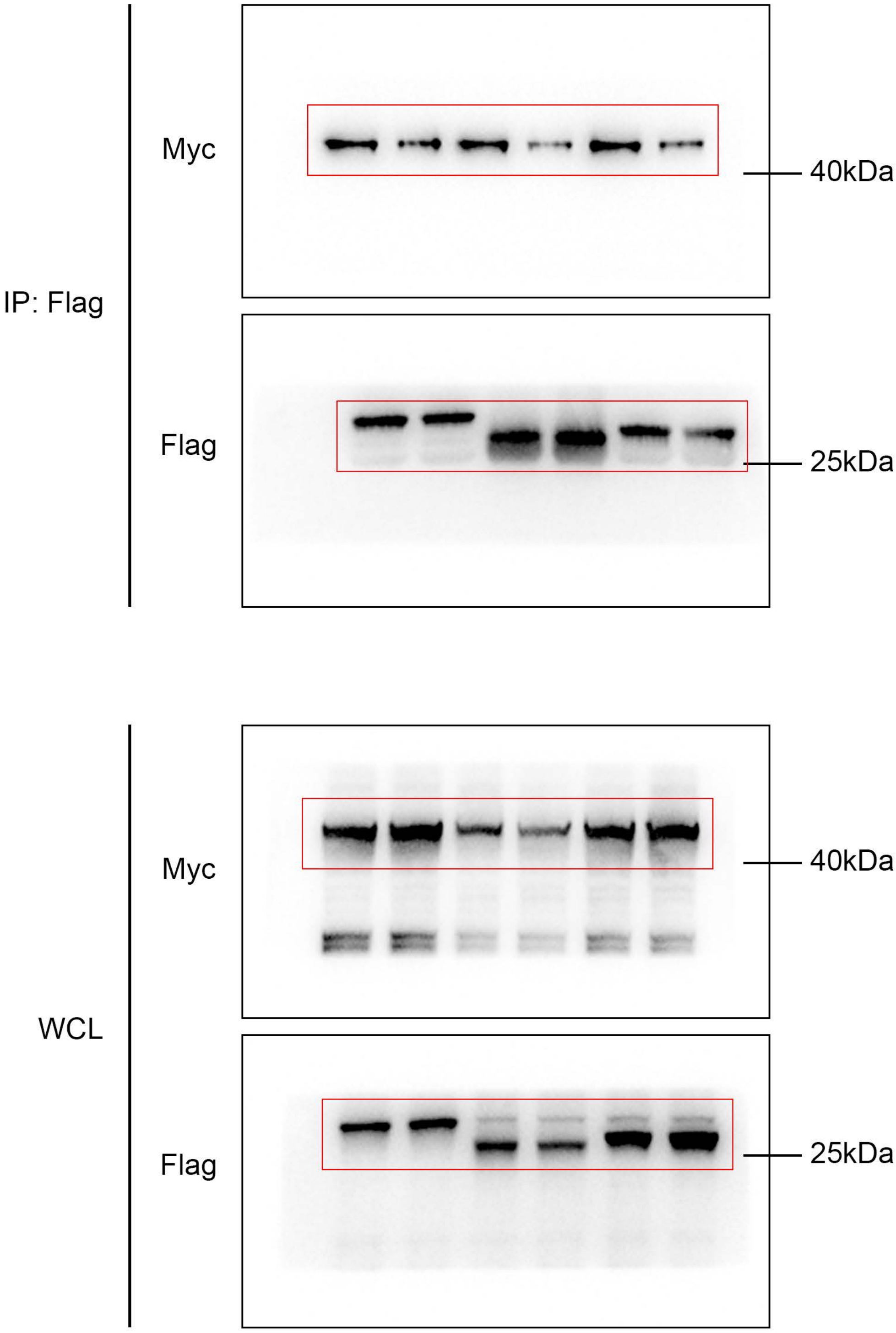

Fig. 5f

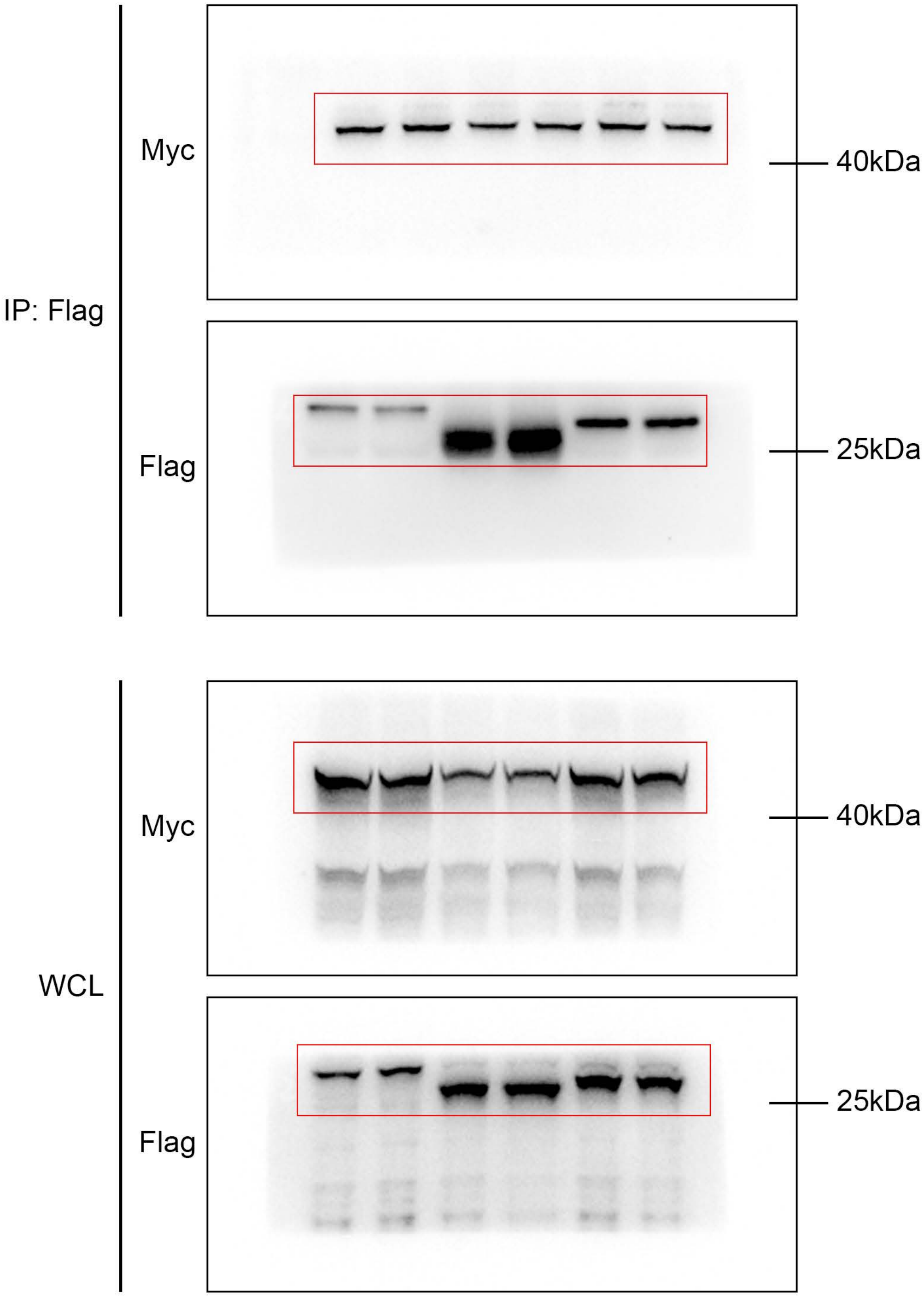

Fig. 5g

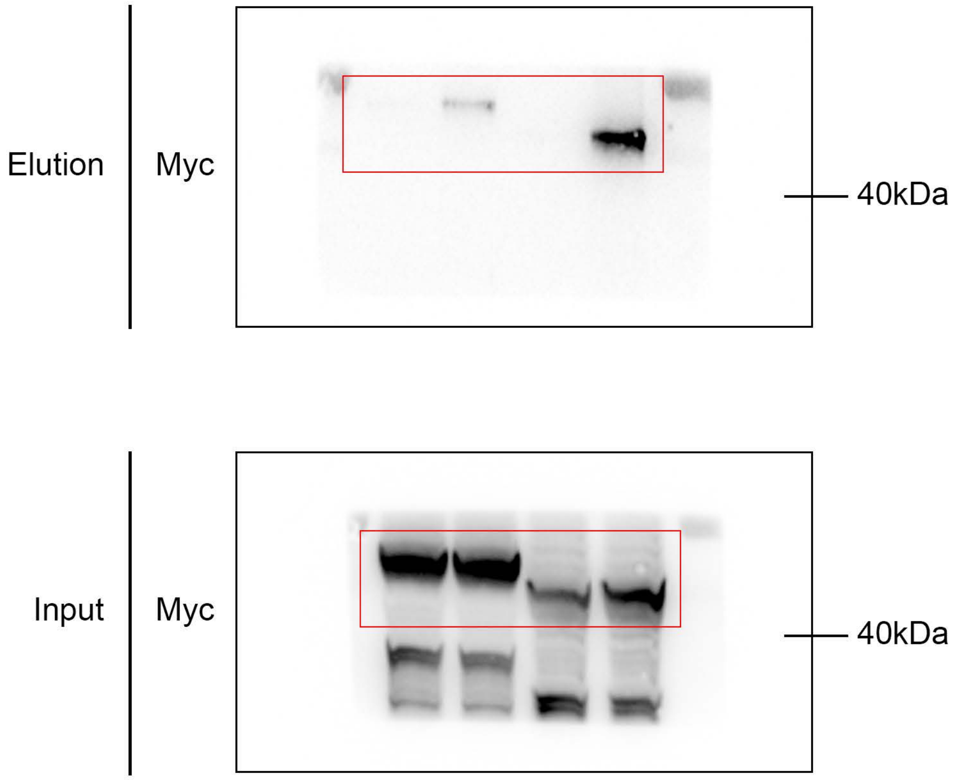
Elution
Myc
40kDa
Input
Myc
40kDa

Fig. 5h

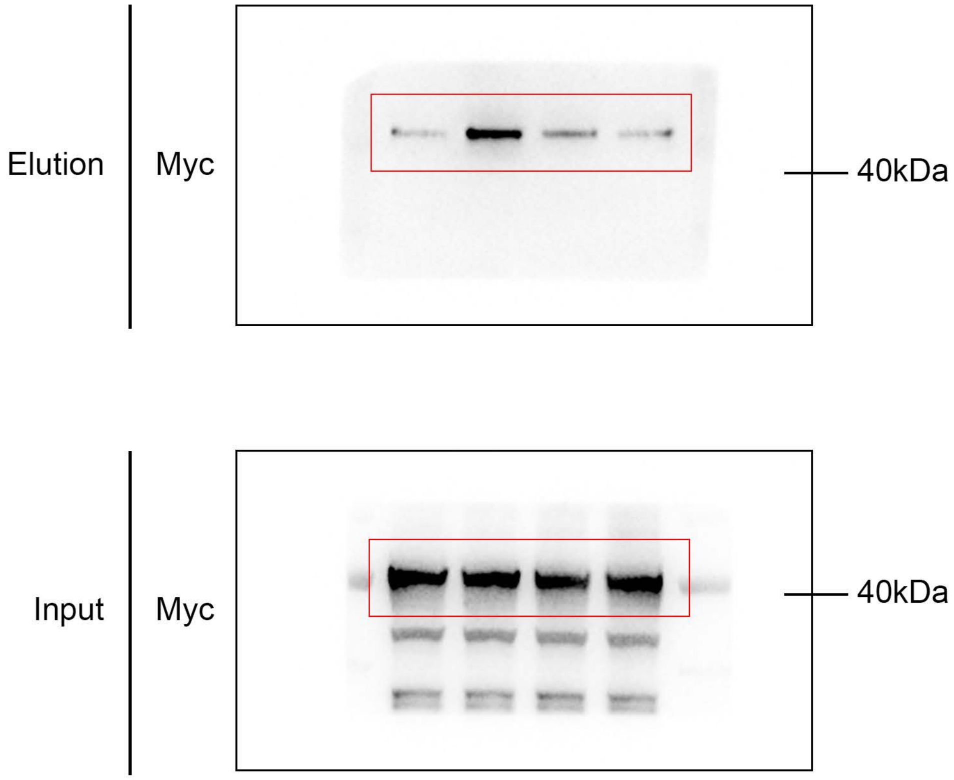
Elution
Myc
40kDa
Input
Myc
40kDa

Fig. 5i

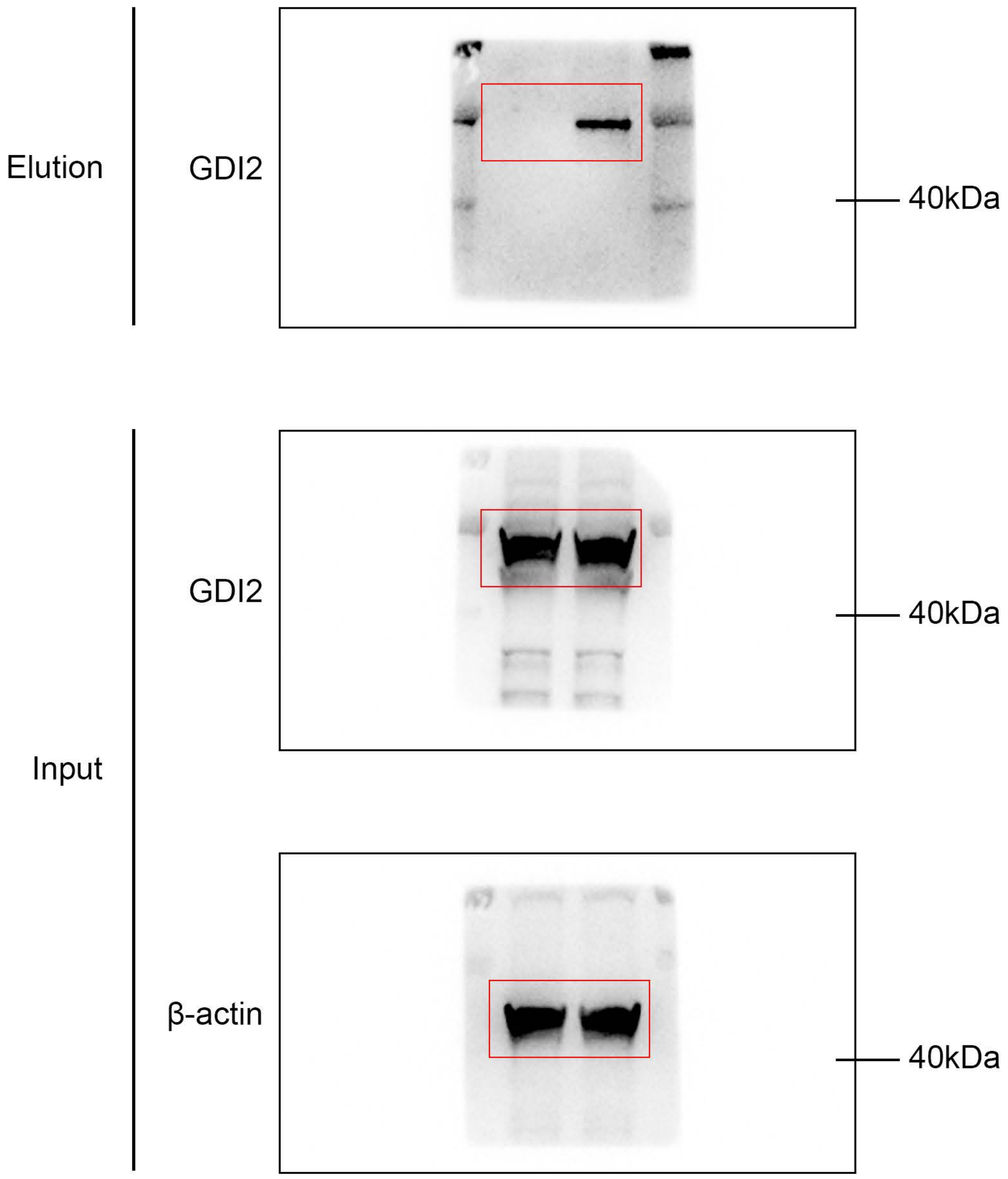

Fig. 5l

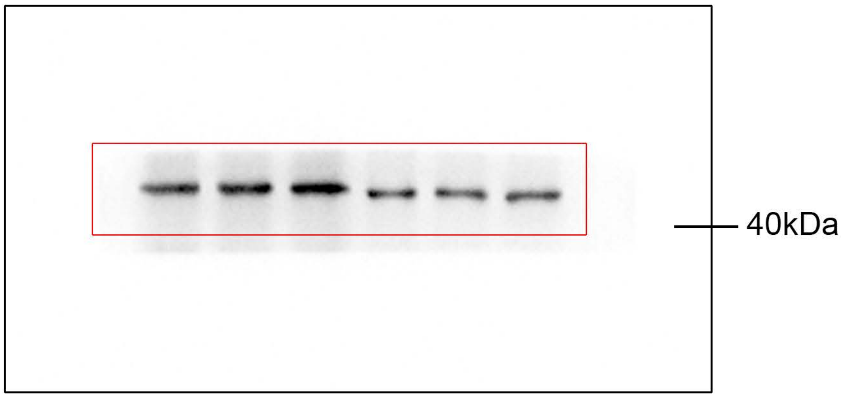

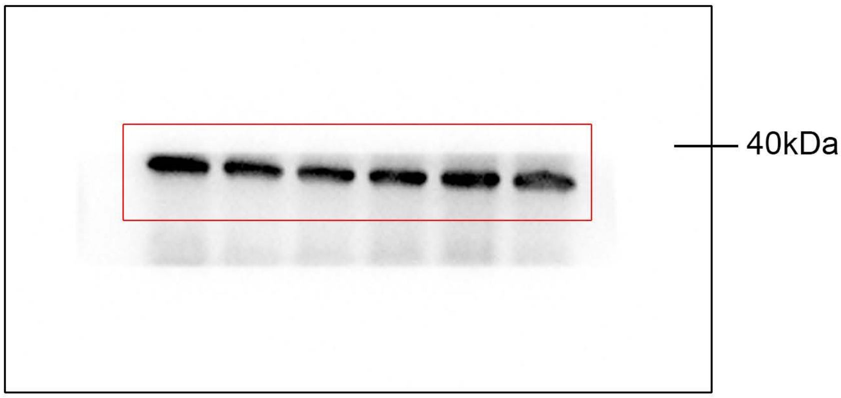

Fig. 6i

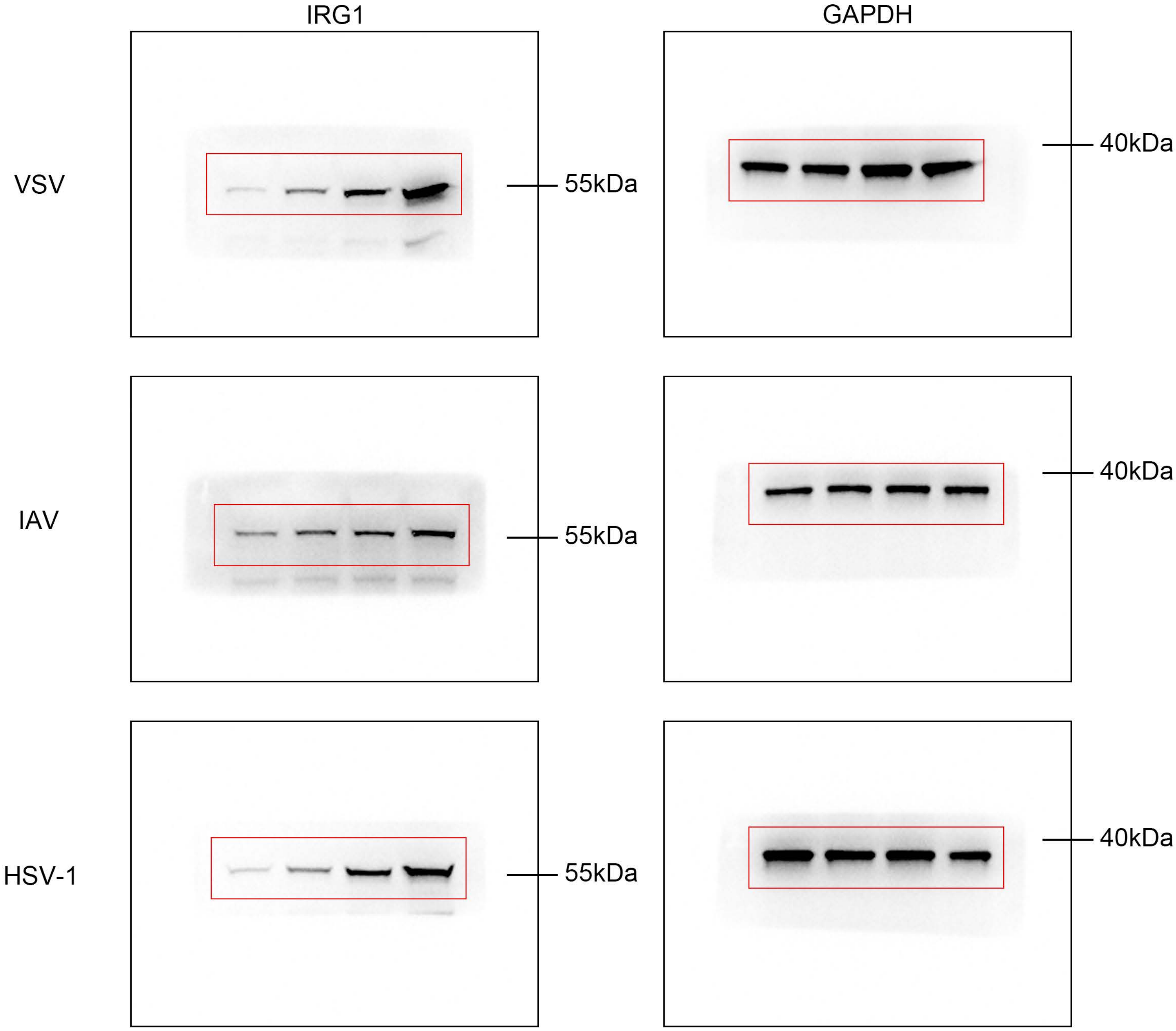

Supplementary Fig.2k

IRG1

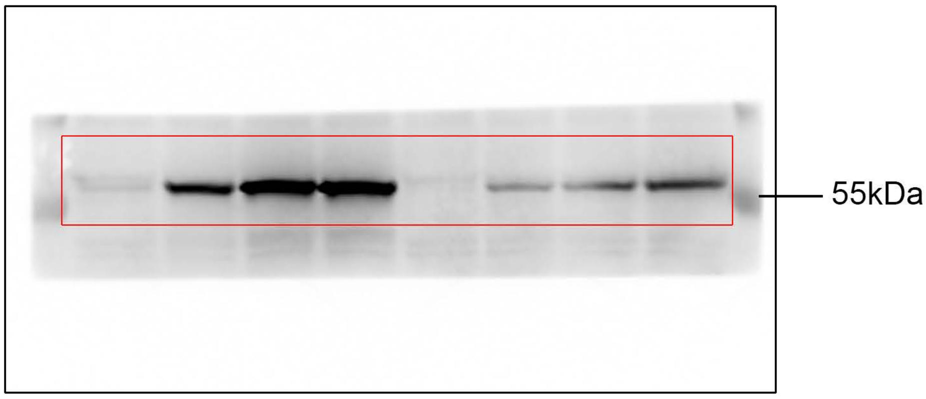

55kDa

GAPDH

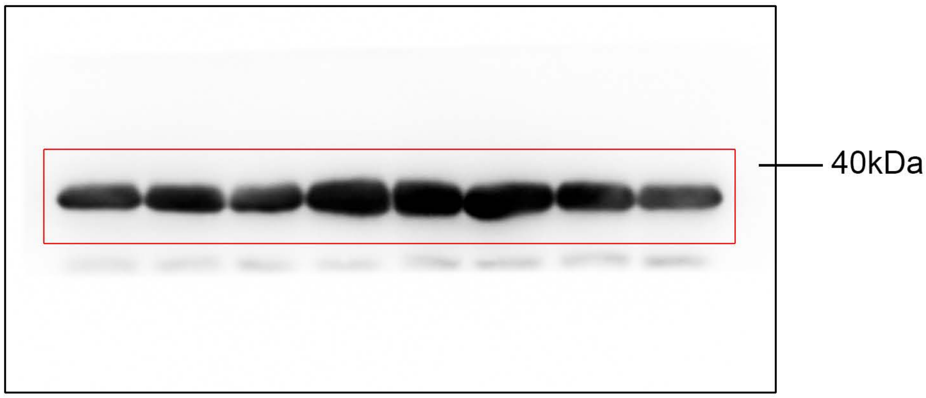

40kDa

Supplementary Fig. 3e

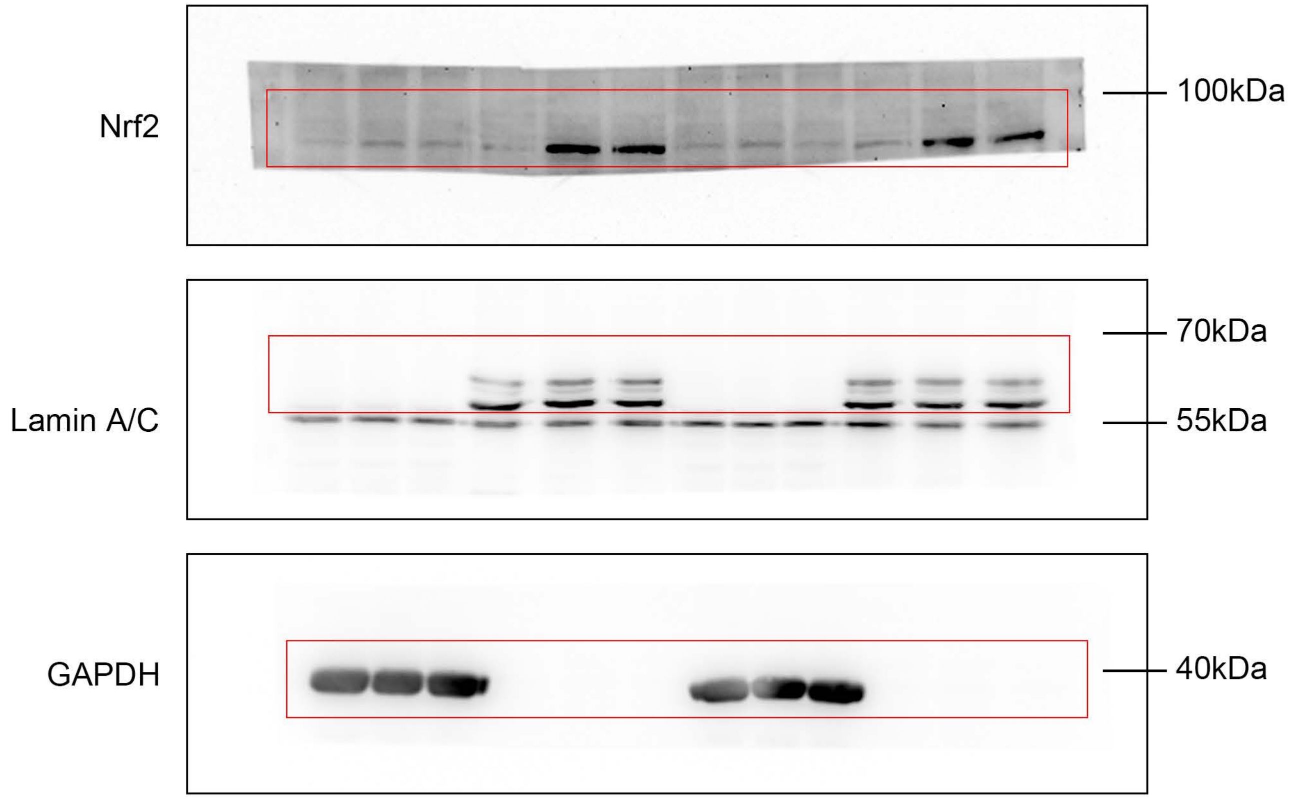

Supplementary Fig. 3g

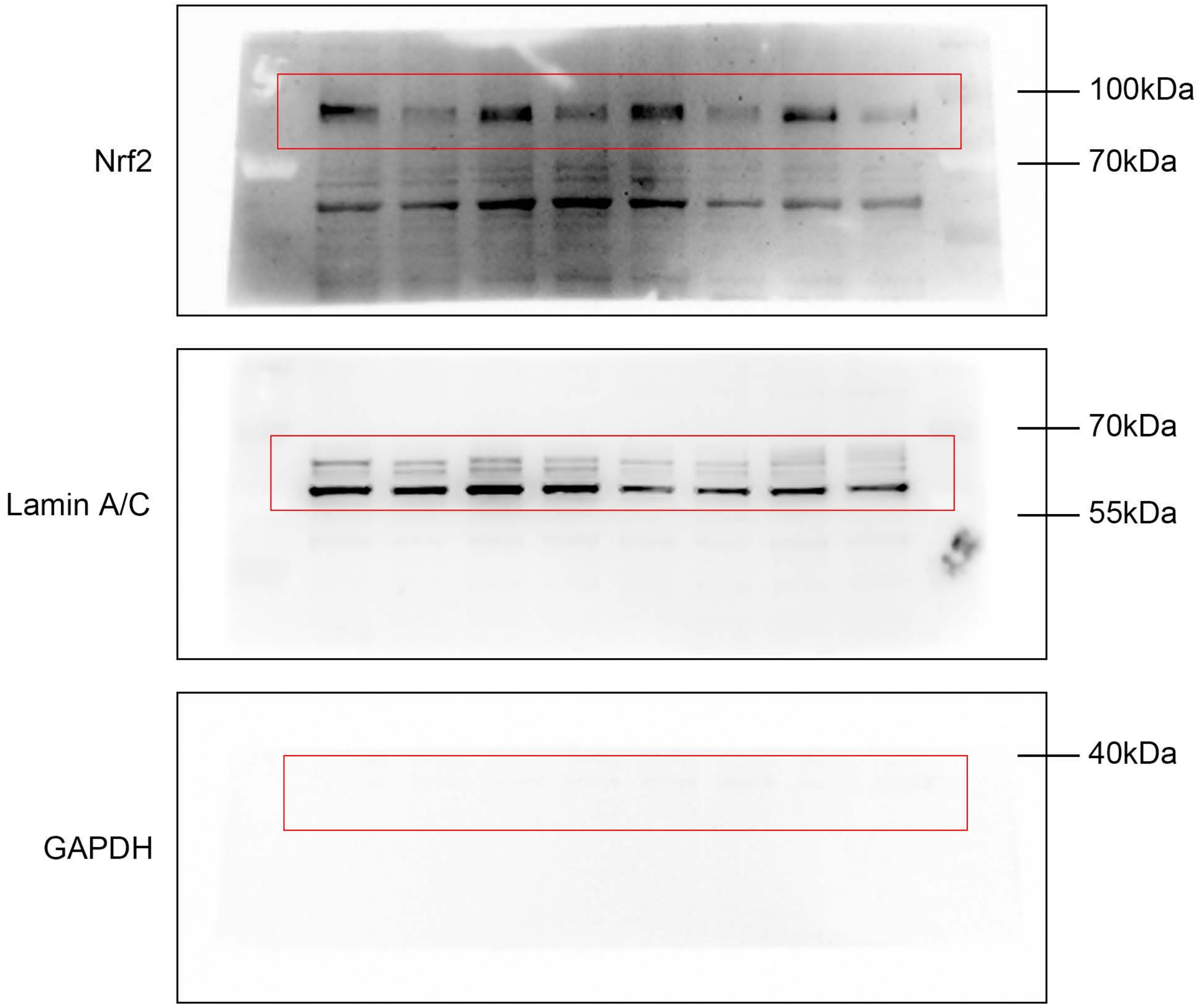

Supplementary Fig. 6a

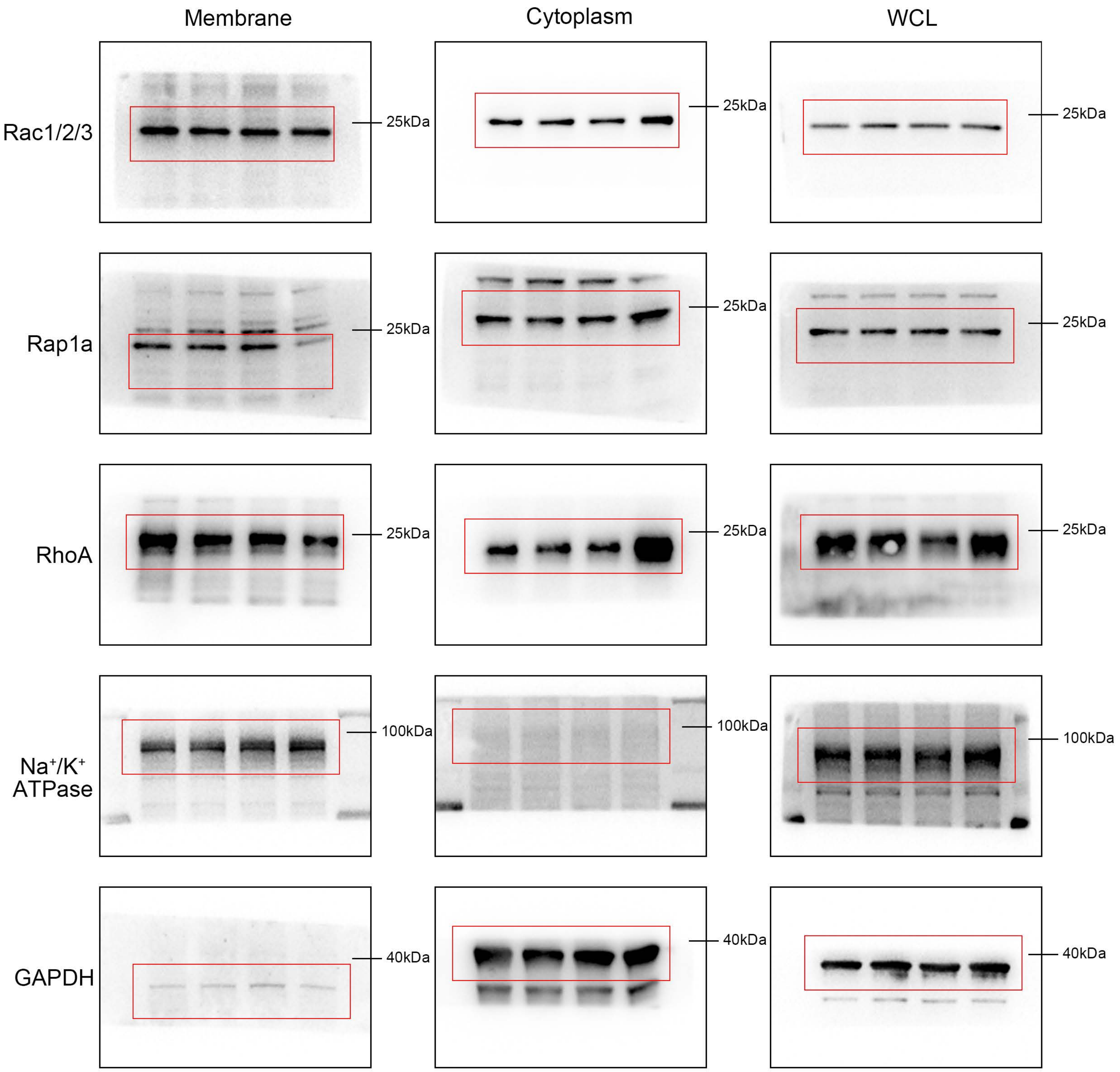

Supplementary Fig. 6c

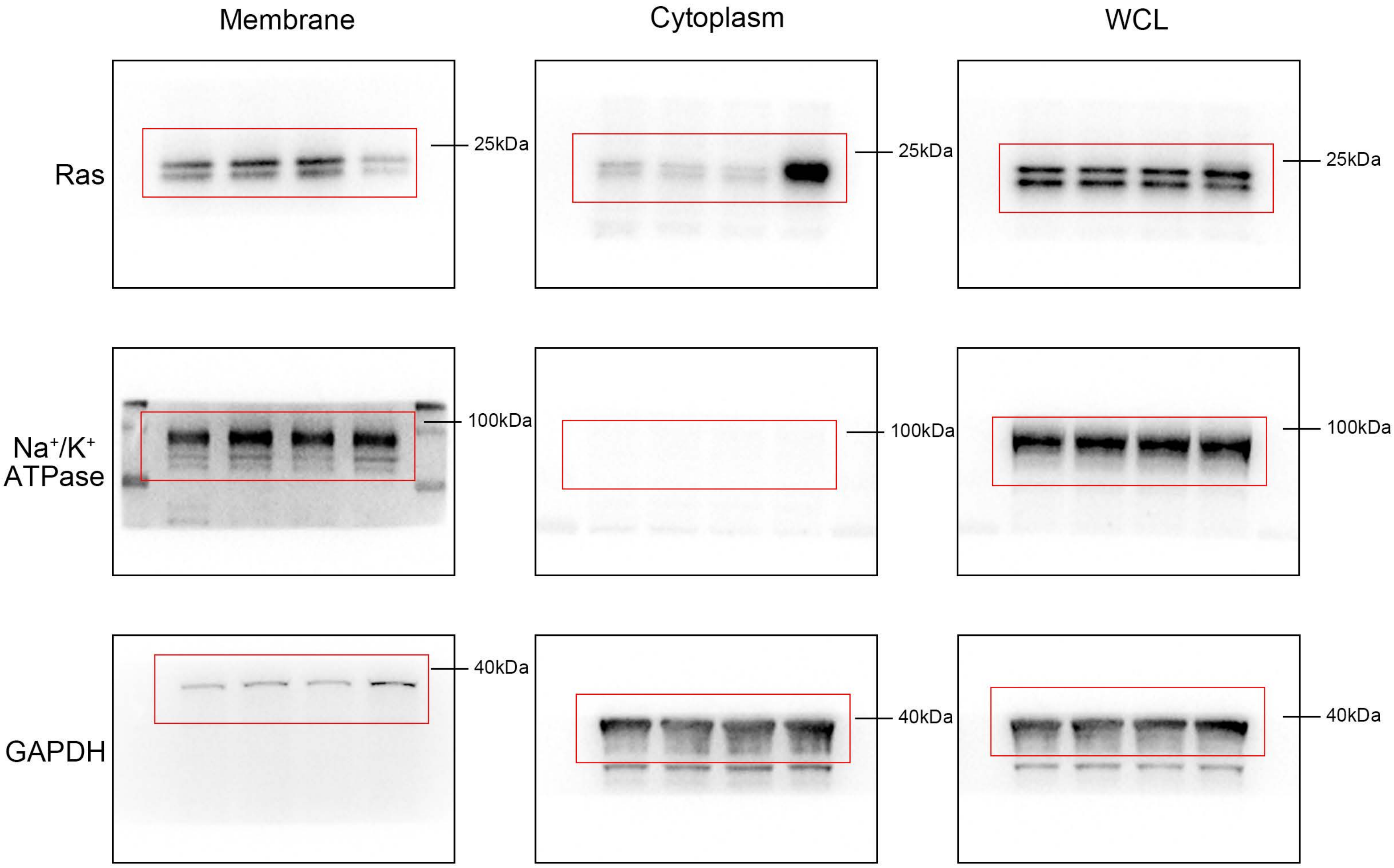

Supplementary Fig. 7a

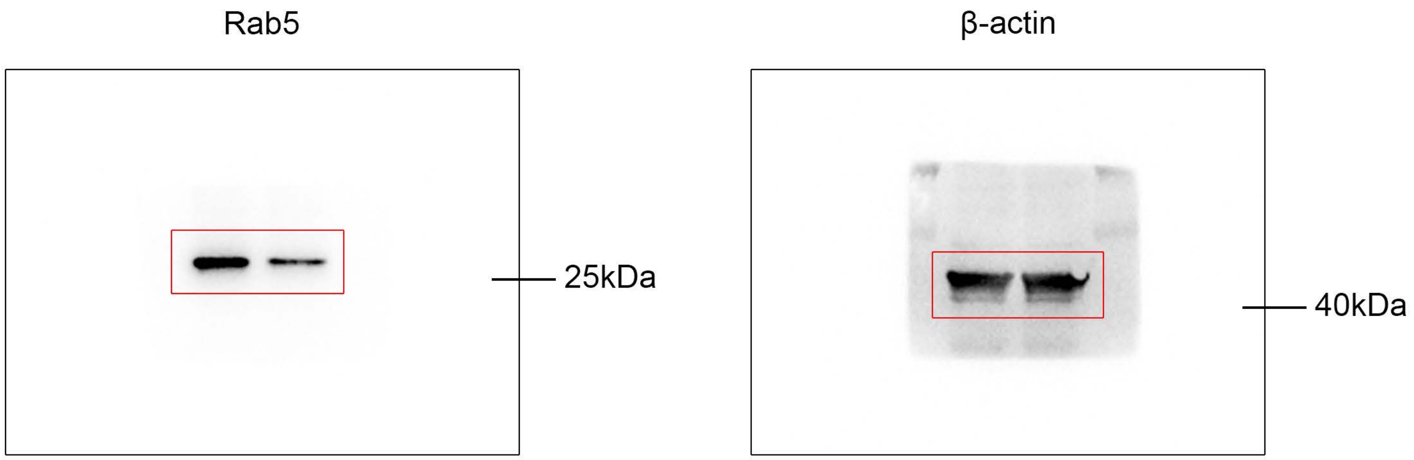

Supplementary Fig. 7b

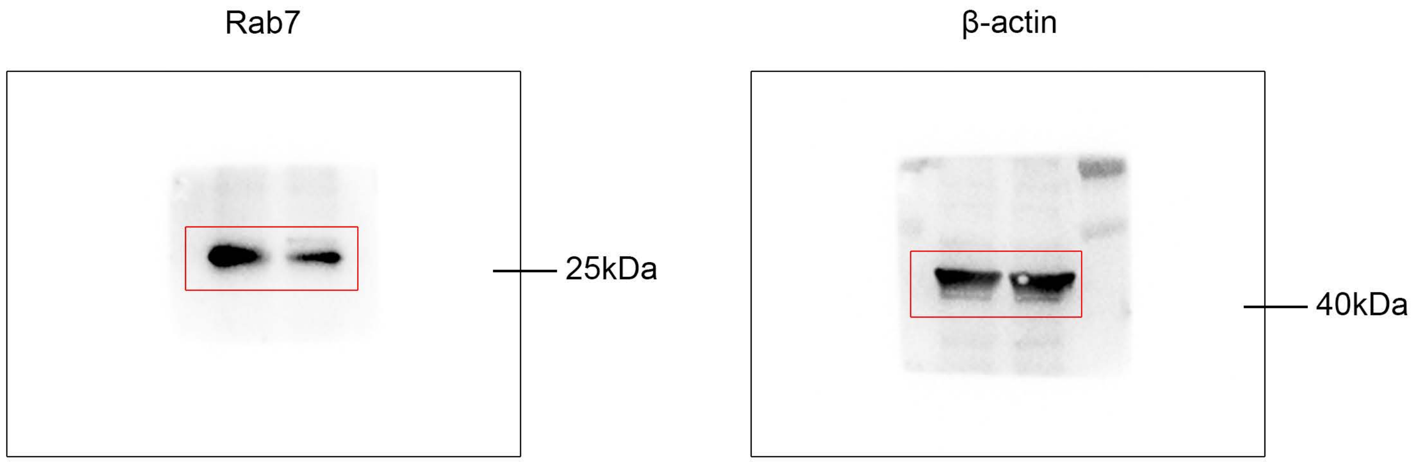

Supplementary Fig. 7c

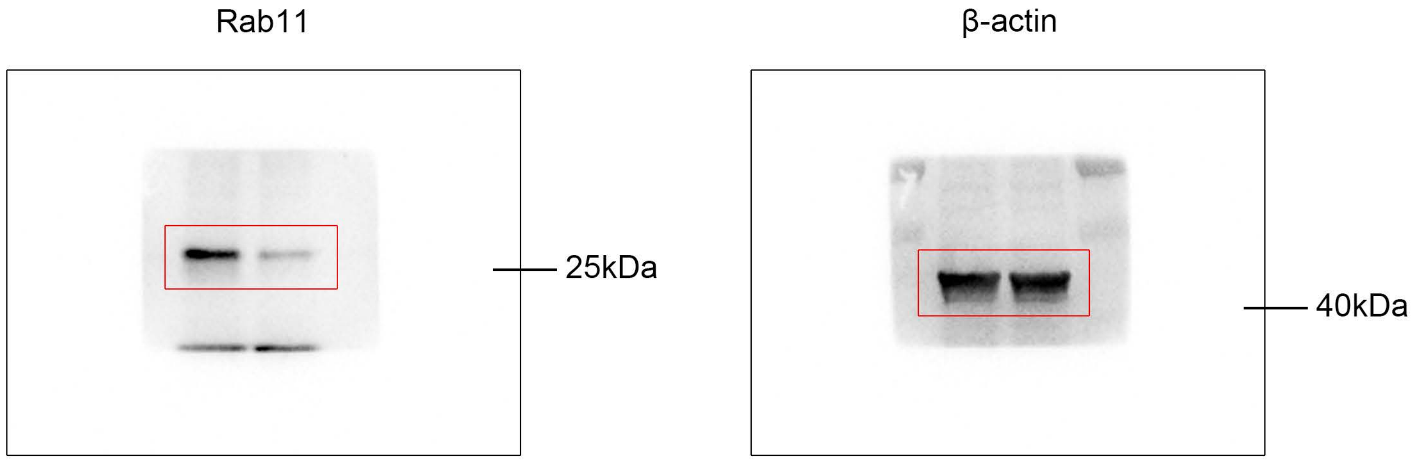

Supplementary Fig. 8a

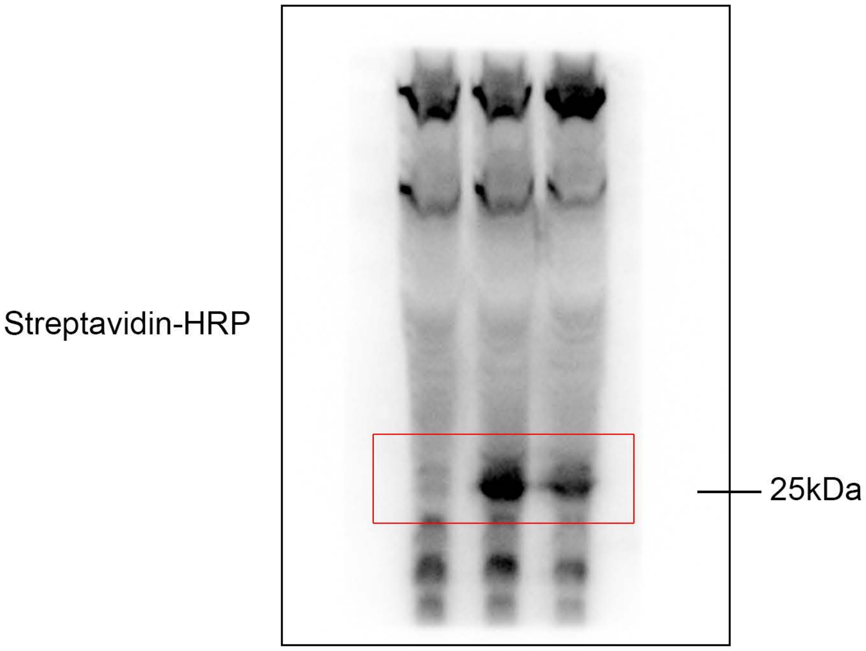

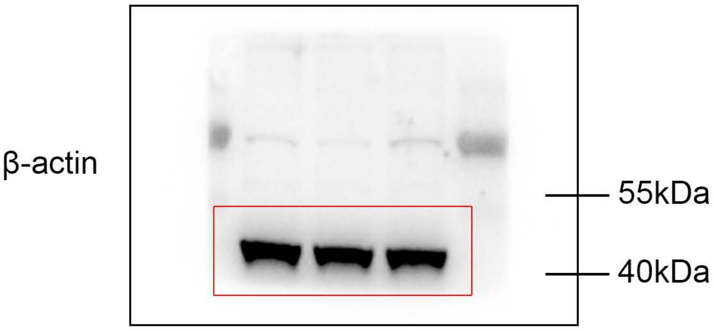

Supplementary Fig. 8b

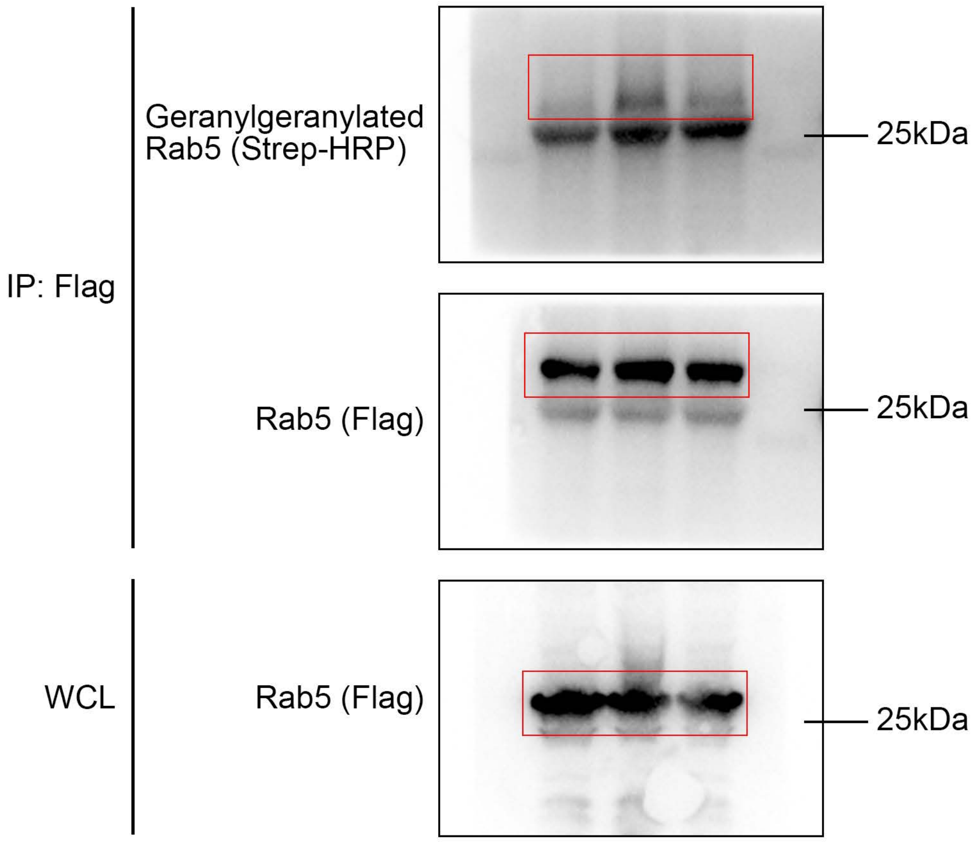

Supplementary Fig 8c

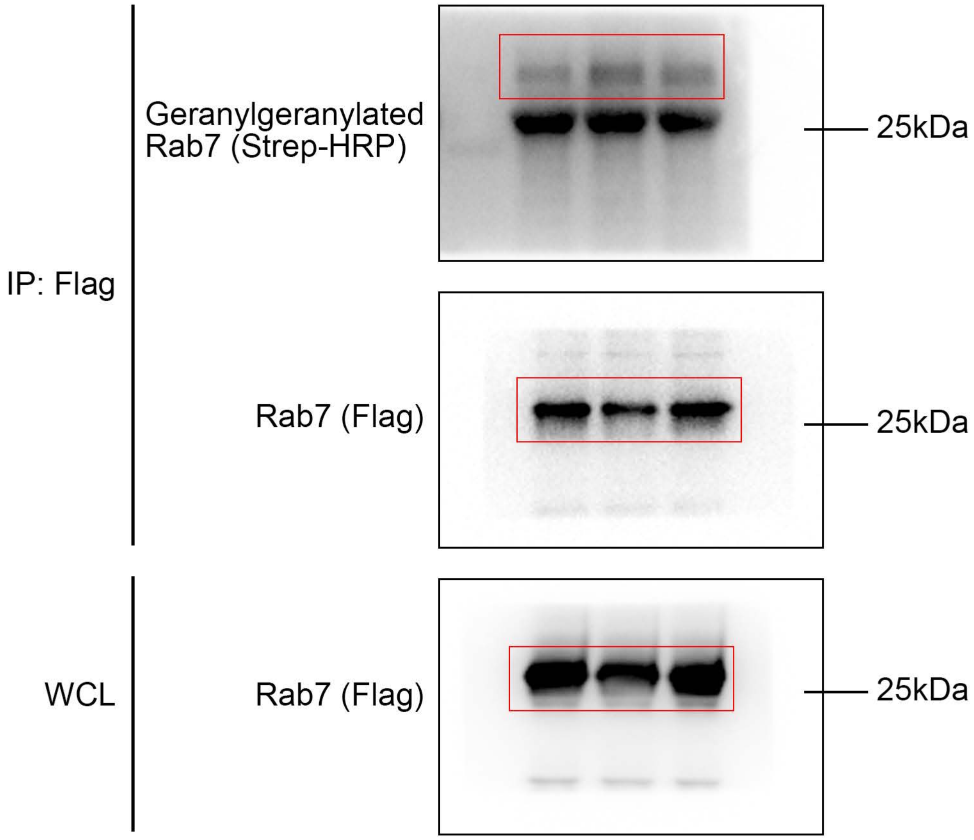

Supplement: Supplementary file 2 — unprocessed original images [file 41392_2024_2077_MOESM2_ESM.pdf]
